# Genome-wide machine learning analysis of anosmia and ageusia with COVID-19

**DOI:** 10.1371/journal.pone.0355832

**Published:** 2026-08-13

**Authors:** Lucas Pietan, Elizabeth Phillippi, Marcelo Melo, Hatem El-Shanti, Brian J. Smith, Benjamin Darbro, Terry Braun, Thomas Casavant

**Affiliations:** 1 Department of Biomedical Engineering, University of Iowa, Iowa City, Iowa, United States of America; 2 Interdisciplinary Graduate Program in Genetics, University of Iowa, Iowa City, Iowa, United States of America; 3 Stead Family Department of Pediatrics, University of Iowa, Iowa City, Iowa, United States of America; 4 Department of Biostatistics, University of Iowa, Iowa City, Iowa, United States of America; 5 Center for Bioinformatics and Computational Biology, University of Iowa, Iowa City, Iowa, United States of America; 6 Department of Electrical and Computer Engineering, University of Iowa, Iowa City, Iowa, United States of America; Taichung Veterans General Hospital, TAIWAN

## Abstract

The COVID-19 pandemic has caused substantial worldwide disruptions in health, economy, and society, manifesting symptoms such as loss of smell (anosmia) and loss of taste (ageusia), that can result in prolonged sensory impairment. Establishing the host genetic etiology of anosmia and ageusia in COVID-19 will aid in the overall understanding of the sensorineural aspect of the disease and contribute to possible treatments or cures. By using human genome sequencing data from the University of Iowa (UI) COVID-19 cohort (N = 187) and the National Institute of Health All of Us (AoU) Research Program COVID-19 cohort (N = 947), we investigated the genetics of anosmia and/or ageusia by employing feature selection techniques to construct a novel variant and gene prioritization pipeline, utilizing machine learning methods for the classification of patients. Models were assessed using a permutation-based variable importance (PVI) strategy for final prioritization of candidate variants and genes. The highest held-out test set area under the receiver operating characteristic (AUROC) curve for models and datasets from the UI cohort was 0.735 and 0.798 for the variant and gene analysis respectively and for the AoU cohort was 0.687 for the variant analysis. Our analysis prioritized several novel and known candidate host genetic factors involved in immune response, neuronal signaling, and calcium signaling supporting previously proposed hypotheses for anosmia/ageusia in COVID-19.

## Introduction

The COVID-19 pandemic caused by the coronavirus SARS-CoV-2 has had a profound impact on the global population, causing unprecedented health, economic, and social disruptions [1,2]. As of May 2024, the World Health Organization (WHO) reported over 775 million confirmed cases and more than 7 million deaths worldwide with over 1 million deaths in the United States [3,4]. COVID-19 presents with a variety of symptoms, ranging from mild respiratory issues to severe pneumonia and multi-organ failure [5]. Among the diverse manifestations of COVID-19, the loss of smell (anosmia) and loss of taste (ageusia) are notable symptoms, affecting around 50% of infected individuals in western countries [6]. While many recover these senses within weeks, a considerable number experience prolonged or even permanent deficits, which can significantly impact quality of life [7].

Multiple biological hypotheses have been proposed to explain the causes of anosmia and ageusia in COVID-19 patients. The most probable cause of the loss of taste with COVID-19 is the viral invasion of gustatory nerves, taste receptor cells, and the olfactory epithelium. Other proposed contributing factors include an increase in pro-inflammatory cytokines leading to damage of associated cells and elevated or decreased levels of gene and protein factors, such as angiotensin II [7,8]. For the loss of smell, a primary hypothesis is the death of support cells for olfactory receptor neurons, affecting neuronal function by altering the olfactory epithelium mucus and disrupting the olfactory cilia [9,10]. Another well-supported hypothesis proposes that the host immune response impacts olfactory receptor neuron function by downregulating gene expression for receptors and signaling molecules, alongside inflammation and damage to the olfactory epithelium and olfactory receptor neurons through cytokine activity [9,11]. Most proposed biological causes of anosmia and ageusia in COVID-19 are not mutually exclusive and the overall etiology is most likely a combination of factors. The difference between acute or long-term anosmia and ageusia symptoms have also been investigated and may have shared or distinct mechanisms [10].

Regarding the molecular mechanisms and specific genes and proteins involved in anosmia and ageusia in COVID-19, immune factors such as Interleukin-6 have been implicated [12]. The largest genome-wide study to date identified 28 variants within the *UGT2A1/UGT2A2* (ENSG00000173610, ENSG00000271271) locus significantly associated with loss of smell or taste in COVID-19 [13]. However, the genetic mechanisms underlying COVID-19-related anosmia and ageusia have yet to be completely explained.

Machine learning (ML) methods have become widely applied in healthcare research, contributing significantly to our understanding of numerous diseases across various disciplines [14]. ML models offer the advantage of simultaneously evaluating multiple features, identifying patterns and interactions within the data undetected by human observation or traditional analytical methods, making them particularly suitable for analyzing genomic data. Machine learning methods have been applied to several areas within genetics, including transcriptomics, epigenomics, and variant discovery [15–17]. Within common, complex trait genomic analyses, the traditional approach involves conducting a genome-wide association study (GWAS) using linear or logistic regression to identify individually associated variants with the phenotype. Significant variants identified in the GWAS are then combined to develop polygenic risk scores (PRS). ML methods have been utilized within the GWAS and PRS analyses to perform a multivariate assessment, increase power of the analysis, and prioritize the significant findings [18–20]. ML methods have also been applied with whole genome/exome sequencing (WGS/ES) and genotyping data, where genomic variants are initially filtered based on pathways or previously associated regions, or used to create gene-region features [21,22].

Feature selection strategies and pipelines have been applied with success to whole genome/exome sequencing data with ML methods to find and prioritize variants and genomic features [23]. Classical statistical filtering strategies, such as chi-squared test filters, have been employed for feature selection with model training and classifying Crohn Disease [24]. Additionally, the Conditional Mutual Information Maximization (CMI) method has been used for selecting features for classifying several diseases [25].

Variable or feature importance analysis is a critical aspect of ML, particularly in medical research and genetics, where the potential for researchers to gain the most insight lies in understanding the utilization of features within the models [26]. When performed correctly, feature selection and model evaluation can produce unbiased results. Permutation-based variable importance (PVI) or feature importance assesses the contribution of each feature in a predictive model and can be assessed across ML models, identifying the features that are significantly influencing predictive performance and are contributing the most important information [27,28].

In this study, we assessed two genome sequencing cohorts for variants and genes associated with the development of anosmia and/or ageusia symptoms in COVID-19. We examined novel feature selection pipelines to construct datasets for ML model to be trained and then tested on held-out test sets to prevent overfitting. A variable importance analysis was leveraged to enhance the interpretability of the models and prioritize the most relevant genetic factors. With this strategy, we found association of several novel genetic factors as well as several known factors including variants and genes involved in immune response, neuronal signaling, and calcium signaling that align with well supported hypothesis in the field.

## Results

### University of Iowa cohort

The University of Iowa (UI) cohort included a total of 187 individuals, with 9 reporting only a loss of smell, 8 reporting only a loss of taste, and 87 reporting a loss in smell and taste. In total, we had 96 cases and 91 controls for the loss of smell phenotype, 95 cases and 92 controls for the loss of taste phenotype, and 104 cases and 83 controls for the loss of smell and/or loss of taste phenotype (S1 Table). Statistics for all other symptoms in the cohort were consistent with previously reported COVID-19 symptoms in the population. The assessment of covariates revealed no association of sex, age, and age-squared with the response variable for any of the phenotypes and were not included in subsequent analyses (S2 Table).

### University of Iowa cohort - COVID-19 loss of smell

**Fig 1:**
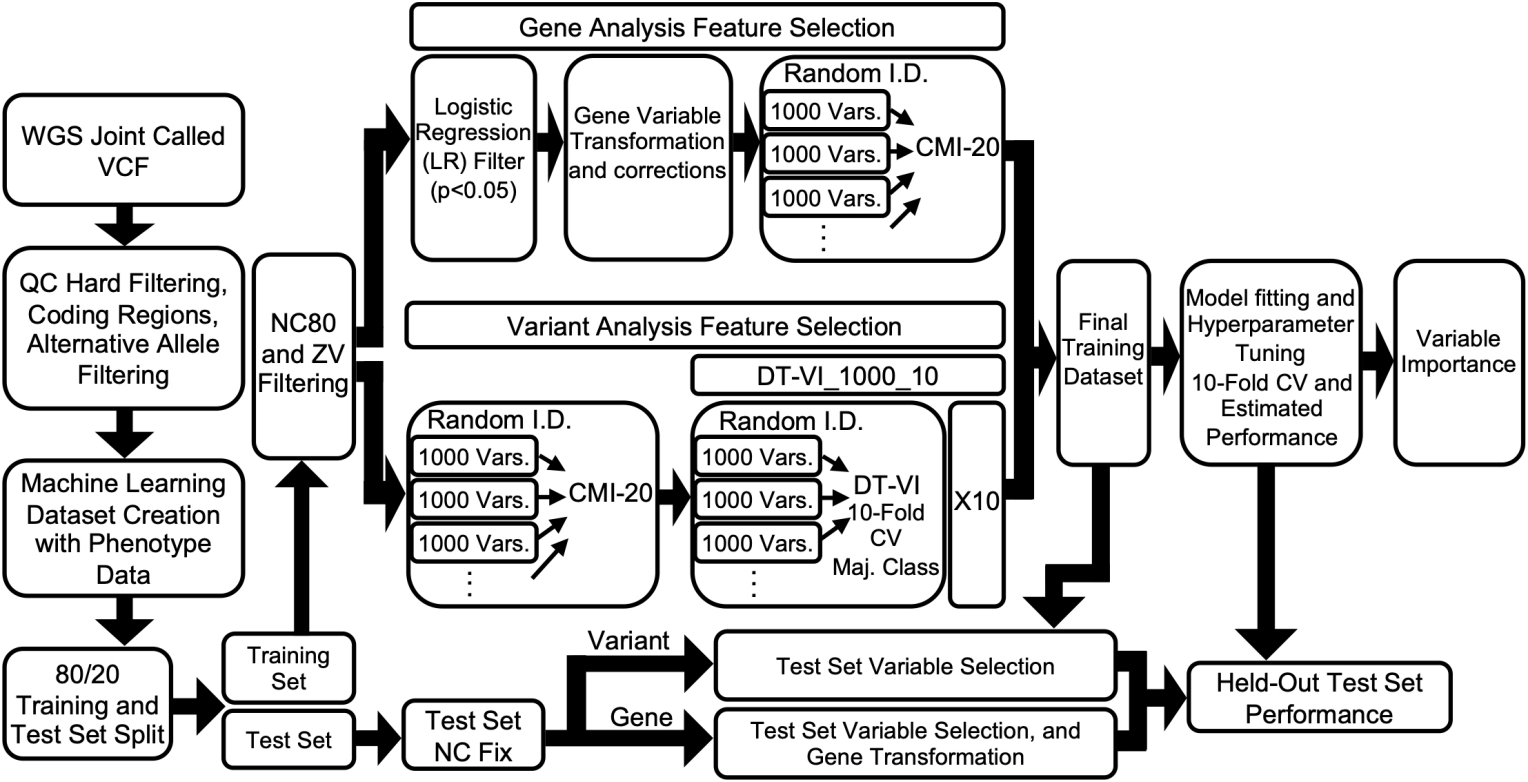
Top performing whole genome sequencing machine learning pipelines for variant and gene analysis. Random I.D. is randomly selected intermediate datasets.

**Fig 2:**
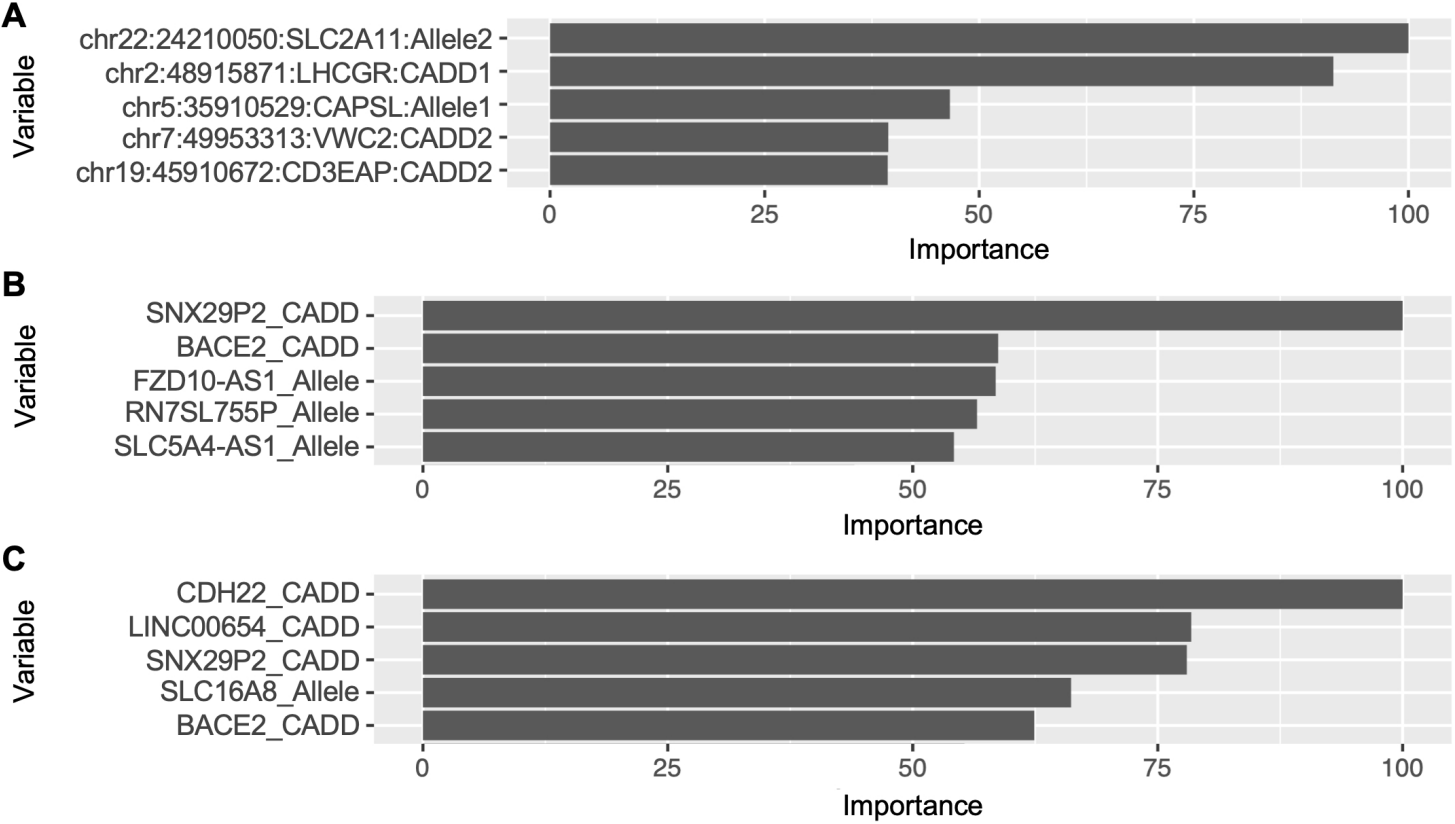
Permutation-based variable importance (PVI) top 5 features for top performing dataset and models for the UI cohort loss of smell analysis. All features included in the model are assessed. (*A*) Variable importance for the extreme gradient boosted tree (XGBTree) model, top performing model for the variant analysis. (*B*) Variable importance for the Elastic Net model, top performing model for the gene analysis according to the accuracy and area under the receiver operating characteristic (AUROC) curve metrics. (*C*) Variable importance for the support vector machine with polynomial kernel function (SVM-P) model, top performing model for the gene analysis according to brier score.

After quality control (QC) filtering and exclusion of variants with multiple alternative alleles the dataset consisted of 9,598,997 variants (38,395,988 features). Our initial examination was on the loss of smell phenotype, where we developed and optimized the feature selection protocols and pipelines. Preliminary testing of our initial filtering pipeline consisting of Conditional Mutual Information-5 (CMI-5), logistic regression (LR), and decision tree variable importance_1000_1 (DT-VI_1000_1) filtering and five ML models with the loss of smell phenotype yielded 1,324 features after filtering and top model performances with the random forest (RF) model with brier score = 0.252 and the support vector machine with polynomial kernel function (SVM-P) model with 58.2% accuracy and 0.577 area under the receiver operating characteristic (AUROC) curve on the held-out test set (S3 Table). Additional filtering of variants within non-coding regions of the genome yielded a dataset of 300,945 variants (1,203,782 features). We saw an increase in performance with this dataset using the same initial filtering pipeline, CMI-5, LR, and DT-VI_1000_1, yielding 47 features with top performances on the held-out test set with the Lasso (accuracy = 73.0%, AUROC curve = 0.682) and RF (brier score = 0.236) models (Table 1, S4 Table). Outside of our initial pipeline to determine the viability of our approach, we extensively examined other feature selection strategies and parameter thresholds. All performance results and trends in performance for all variant and gene feature selection strategies and pipelines are included in the supplemental documentation (S5 and S6 Tables). The top performing variant feature selection pipeline was using the combination of filtering algorithms CMI-20, DT-VI_1000_10 (Fig 1), resulting in 1,015 features (S7 Table) and the extreme gradient boosted tree (XGBTree) model as the top performing model (accuracy = 75.7% [61.9%, 89.5%], brier score = 0.216 [0.113, 0.336], AUROC curve = 0.735 [0.561, 0.909]) on the held-out test set (Table 1, S8 Table). The top two most important features from PVI for the XGBTree model are from variants in the *SLC2A11* (ENSG00000133460) and *LHCGR* (ENSG00000138039) genes (Fig 2A, Table 2, S9 Table). The top performing pipeline for the gene feature analysis was LR (p-value threshold = 0.05), gene feature transformation with the sample allele frequency correction, CMI-20 (Fig 1). This pipeline resulted in 107 gene features (S7 Table) and Elastic Net (accuracy = 81.1% [68.5%, 93.7%], AUROC curve = 0.798 [0.643, 0.954]) and SVM-P (brier score = 0.192 [0.119, 0.268], AUROC curve = 0.798 [0.648, 0.948]) as the top performing models on the held out-test set (Table 1, S10 Table). Top features of importance were from the *SNX29P2* (ENSG00000271699) and *CDH22* (ENSG00000149654) genes for the Elastic Net and SVM-P models respectively, as well as sharing several top features of importance including features from the *SNX29P2*, *BACE2* (ENSG00000182240), and *SLC16A8* (ENSG00000100156) genes (Figs 2B,C, Table 2, S11 Table). All results for all variant and gene feature selection strategies and pipelines are included in the supplemental documentation (S5 and S6 Tables).

**Table 1:** UI top feature selection pipeline datasets for the loss of smell phenotype held-out test set performance.

CMI-5, LR, and DT-VI_1000_1										
	DT	RF	Lasso	NB	SVM-L	SVM-P	SVM-RB	XGBTree	Elastic Net	Log Reg
Brier Score	0.321	0.236	0.268	0.363	0.309	0.331	0.319	0.339	0.285	0.319
Accuracy	56.8%	67.6%	73.0%	62.2%	54.1%	64.9%	62.2%	56.8%	64.9%	62.2%
AUROC Curve	0.539	0.665	0.682	0.592	0.676	0.619	0.637	0.592	0.658	0.637
CMI-20, DT-VI_1000_10										
Brier Score	0.293	0.223	0.273	0.323	0.274	0.247	0.256	0.216	0.279	0.256
Accuracy	51.4%	67.6%	64.9%	67.6%	56.8%	67.6%	43.2%	75.7%	64.9%	43.2%
AUROC Curve	0.503	0.731	0.679	0.628	0.622	0.685	0.313	0.735	0.655	0.313
LR (p-value threshold = 0.05), gene feature transformation with the sample allele frequency correction, CMI-20										
Brier Score	0.251	0.213	0.205	0.350	0.206	0.192	0.197	0.245	0.193	0.197
Accuracy	59.5%	70.3%	67.6%	64.9%	70.3%	73.0%	75.7%	67.6%	81.1%	75.7%
AUROC Curve	0.588	0.699	0.746	0.678	0.749	0.798	0.772	0.684	0.798	0.772

**Table 2:** Summary of machine learning (ML) analysis results for variants and genes in cohort and phenotype top performing datasets.

Cohort	Phenotype	Analysis	Feature Selection Pipeline	Number of Filtered Dataset Features	PVI Top Feature(s)	Variant/Gene Information
UI	Loss of Smell	Variant	CMI-20, DT-VI_1000_10	1,015	chr22:24210050:SLC2A11:Allele2	rs4428104, A>C, intron_variant, CADD = 1.466, Cases 74, Controls 42
UI	Loss of Smell	Gene	LR (p-value threshold = 0.05), gene feature transformation with the sample allele frequency correction, CMI-20	107	SNX29P2_CADD	SNX29P2, number of variant features = 3, Cases 3.47, Controls 6.06
UI	Loss of Taste	Variant	CMI-5	5,165	chr10:5037718:AKR1C2:Allele1	rs78396968, A>G, intron_variant, CADD = 4.417, Cases 16, Controls 19
UI	Loss of Taste	Gene	LR (p-value threshold = 0.05), gene feature transformation with the directional correction, CMI-20	103	GRIN3A_CADD, Y_RNA_Allele	GRIN3A, number of variant features = 4, Cases 35.99, Controls 30.61; Y_RNA, number of variant features = 21, Cases 12.89, Controls 10.87
UI	Loss of Smell and/or Taste	Variant	CMI-5, LR, and DT-VI_1000_1	56	chr11:4621639:TRIM68:CADD2, chr22:39631768:PDGFB:CADD2	rs2231975, C > T, missense_variant, C442Y, CADD = 0.287, Cases 2, Controls 16; rs17303681, G > T, intron_variant, CADD = 2.866, Cases 4, Controls 11
UI	Loss of Smell and/or Taste	Gene	LR (p-value threshold = 0.05), gene feature transformation with the sample frequency correction, CMI-20 pipeline	102	GNAO1_CADD	GNAO1, number of variant features = 8, Cases 5.44, Controls 2.54
AoU	Loss of Smell and/or Taste	Variant	Combioned CMI-5, LR (chi-squared test/F-test), DT-VI_1000_1	19	chr9:136670216:EGFL7:Allele2	rs2297538, G>A, missense_variant, V153I, CADD = 10.15, Cases 180, Controls 157
AoU	Loss of Smell and/or Taste	Gene	LR (p-value threshold = 0.05), gene feature transformation with no correction, CMI-20	171	AHNAK2_Allele	AHNAK2, number of variant features = 25, Cases 0.29, Controls 0.14

Variant/Gene Information case and control values are counts of samples with the alternative allele for variant features and averages for gene features.

### University of Iowa cohort - COVID-19 loss of taste

The top performing feature selection pipelines established during the loss of smell phenotype analysis were used to investigate the loss of taste phenotype and the loss of smell and/or loss of taste phenotype. Top results for the loss of taste phenotype for the variant analysis was the single CMI-5 filter and the decision tree (DT) model (accuracy = 62.2% [46.5%, 77.8%], brier score = 0.245 [0.199, 0.301], AUROC curve = 0.647 [0.476, 0.818], S12 Table) fit to 5,165 features (S7 Table). PVI for the DT model had the top feature from a variant in the *AKR1C2* (ENSG00000151632) gene (Table 2, S1A Fig, S13 Table). For the gene analysis, the top pipeline was LR (p-value threshold = 0.05), gene feature transformation with the directional correction, CMI-20, yielding 103 candidate features (S7 Table). Top models on the held-out test set were the support vector machine with linear kernel function (SVM-L) (accuracy = 73.0% [58.7%, 87.3%], AUROC curve = 0.709 [0.534, 0.884]) and RF models (brier score = 0.248 [0.205, 0.290]) (S14 Table). The most important feature from the SVM-L model was *GRIN3A* (ENSG00000198785) gene feature and from the RF model was from the Y RNA gene locus. The *snoU13* (ENSG00000238692) gene feature was also shared in top features in both models (Table 2, S1B,C Figs, S15 Table). Model performances for all filtering strategies and datasets for the loss of taste phenotype are included in the supplemental documentation (S16 Table).

### University of Iowa cohort - COVID-19 loss of smell and/or taste

As for the loss of smell and/or loss of taste phenotype, the top variant pipeline was CMI-5, LR, and DT-VI_1000_1, filtering to 56 features (S7 Table) with top model performance on the held-out test set with the RF (accuracy = 64.9% [49.5%, 80.2%], brier score = 0.242 [0.196, 0.290]) and Naïve Bayes (NB) (accuracy = 64.9% [49.5%, 80.2%], AUROC curve = 0.670 [0.487, 0.852]) models (S17 Table). PVI showed the most important feature for the RF model to be from the *TRIM68* (ENSG00000167333) gene and for the NB to be from the *PDGFB* (ENSG00000100311) gene. There was also a shared top important variant feature from the *IL21-AS1* (ENSG00000227145) gene for the RF and NB models (Table 2, S2A,B Figs, S18 Table). The top gene pipeline was the LR (p-value threshold = 0.05), gene feature transformation with the sample frequency correction, CMI-20 pipeline, selecting 102 gene features (S7 Table). The top model performance on the held-out test set was with the XGBTree model (accuracy = 73.0% [58.7%, 87.3%], brier score = 0.206 [0.124, 0.297], AUROC curve = 0.778 [0.616, 0.939], S19 Table). Top feature for the XGBTree model was a *GNAO1* (ENSG00000087258) gene feature (Table 2, S2C Fig, S20 Table). Model performances for all filtering strategies and datasets for the loss of smell and/or taste phenotype are included in the supplemental documentation (S21 Table).

### Intersection of genetic features between UI cohort phenotypes

Comparing the filtered datasets from the top performing pipelines for each phenotype we can assess possible shared genetics between the loss of smell and loss of taste with COVID-19. For overlap of variant features, 73 features were intersected with two of the three datasets for each phenotype and three features were found in all three datasets (chr12:131440935:GPR133:CADD1, chr19:56466227:NLRP8:CADD2, chr20:61472938:TCFL5:CADD2). Breaking the features down by position in the genome, two of the three datasets shared 96 features within the same position in the genome and three feature positions agreed across all three datasets (chr12:131440935, chr19:56466227, chr20:61472938). There were 864 genes intersected with features between two datasets and 6 genes (*C1orf127* (ENSG00000175262)*, DOCK1* (ENSG00000150760)*, GPR133* (ENSG00000111452)*, NLRP8* (ENSG00000179709)*, TCFL5* (ENSG00000101190)*, TRIM68* (ENSG00000167333)) by features of all three datasets. Comparing the intersection of datasets from the gene analysis, 24 features and 28 genes were overlapped in two of the three datasets and the same four features (BCAT1_Allele, CTC-340A15.2_Allele, MSRA_CADD, PARVB_Allele) and four genes were shared by all three datasets (S7 and S22 Tables).

### Testing University of Iowa cohort models on the All of Us cohort

To examine the generalizability of the genetic association found in the UI cohort with loss of smell and loss of taste symptoms, we sought to test the models and selected genomic features on an independent dataset from the All of Us (AoU) Research Program. The AoU cohort included a total of 947 individuals, with 421 reporting a loss of smell or taste with COVID-19. In total, we had 421 cases and 526 controls for our analysis (S23 Table). Testing results from the six top performing datasets with all models from the UI cohort variant and gene analyses with the three phenotypes showed little shared association between datasets. Model performances on the held-out test sets were inferior or equal to a majority class model (accuracy = 55.5%) across all datasets and models with ranges in accuracies = [44.5%, 55.6%], brier scores = [0.251, 0.550], and AUROC curves = [0.448, 0.539] (S24 Table).

### All of Us cohort - COVID-19 loss of smell and/or taste

**Fig 3:**
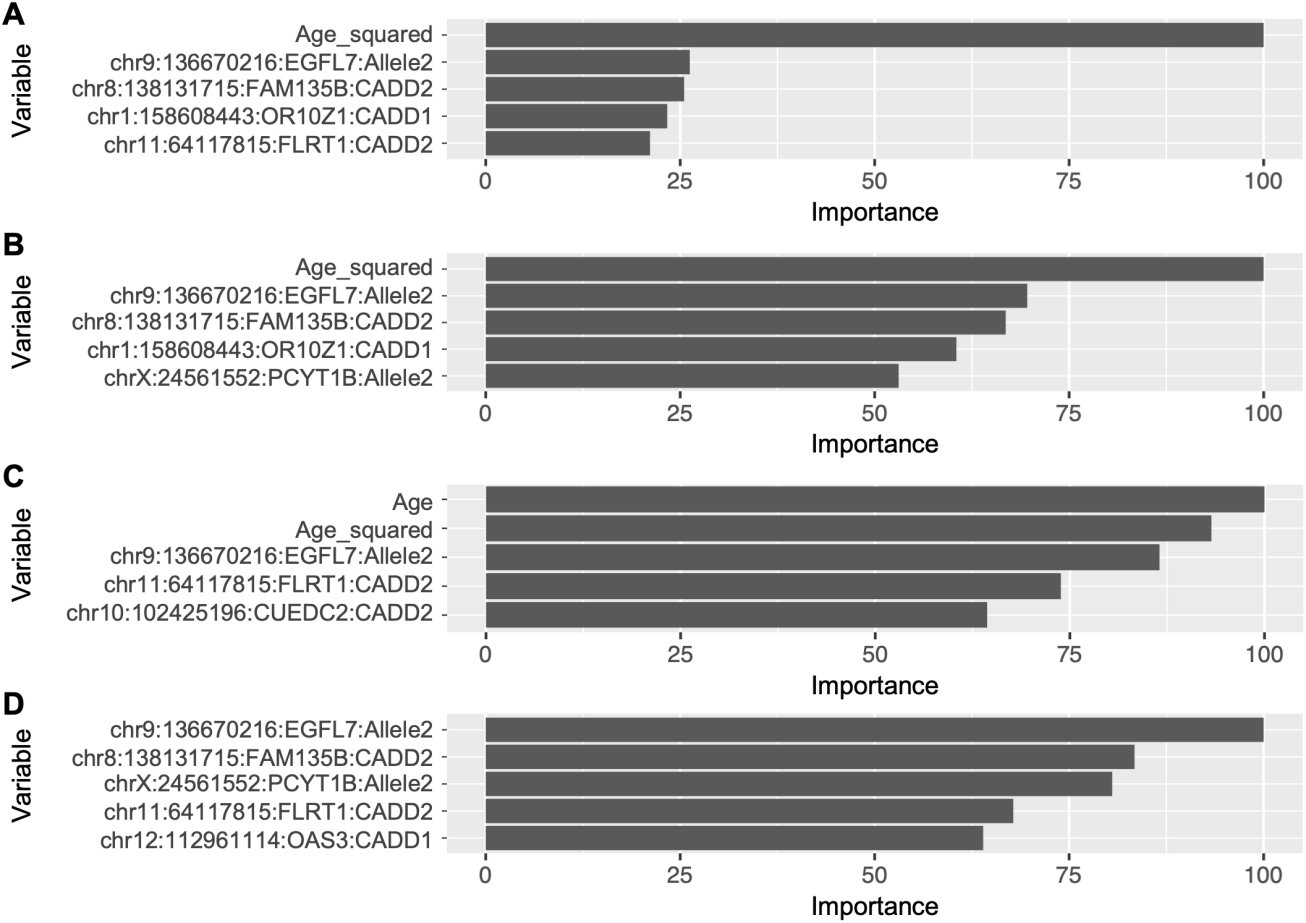
Permutation-based variable importance top 5 features for top performing dataset and models for the AoU cohort variant analysis. All features included in the model are assessed. (*A*) Variable importance for the Lasso model, top performing model for the variant analysis according to the accuracy metric. (*B*) Variable importance for the Elastic Net model (accuracy). (*C*) Variable importance for the RF model (brier score). (*D*) Variable importance for the SVM-RB model (brier score and AUROC curve).

The validation results led us to examine the compatibility and similarities between the two cohorts with principal component analysis (PCA). PCA and identity-by-state distance calculations showed the two cohorts to have more variation in the data due to cohort classification than to case/control classification (S25 Table, S3A-L Figs). An assessment of covariates with the AoU cohort also revealed an association with sex (p-value = 0.000367), age, and age-squared (p-values < 2e-16) covariates with the response variable. We then performed a complete gene and variant machine learning analysis with our established best performing feature selection pipelines on the AoU cohort. After QC filtering, exclusion of variants with multiple alternative alleles, and filtering of variants within non-coding regions of the genome, the dataset consisted of 1,583,701 variants (6,334,804 features). First, we performed the analysis without covariates and then with covariates, as well as a covariate only model. The best performing feature selection pipelines were the CMI-5, LR (chi-squared test), DT-VI_1000_1 and CMI-5, LR (F-Test), DT-VI_1000_1 yielding datasets of 9 and 10 features, respectively. Model performance was similar for both feature sets with the CMI-5, LR (chi-squared test), DT-VI_1000_1 dataset having an accuracy = 61.9% and the CMI-5, LR (F-Test), DT-VI_1000_1 pipeline with an accuracy = 61.4% on the held-out test sets with a NB model (Table 3, S26 Table). A combination of the two datasets (19 features) resulted in similar performance across models (Table 3, S26 Table). Addition of the covariates to all datasets tested led to the overall best performance with the combined dataset with the top models being Lasso (accuracy = 68.8% [62.2%, 75.4%]), Elastic Net (accuracy = 68.8% [62.2%, 75.4%]), RF (brier score = 0.222 [0.193, 0.251]), and SVM-RB (brier score = 0.222 [0.193, 0.252], AUROC curve = 0.687 [0.609, 0.766]) (Table 3, S26 Table). PVI showed a variant feature from the *EGFL7* (ENSG00000172889) gene to be the most important feature across top models as well as the Age and Age-squared covariates impacting model performances (Figs 3A-C, Table 2, S27 Table). Another notable top feature of importance across models is a variant from the *GRM1* (ENSG00000152822) gene. The covariate only models demonstrated slightly stronger performance when considering the metrics brier score and AUROC curve for the held-out test set but weaker performance when considering mean metrics for the 10-fold CV training performance and held-out test set accuracy when compared to the full dataset models (Table 3, S26 Table). For the gene analysis, the LR (p-value threshold = 0.05), gene feature transformation with no correction, CMI-20, selecting 171 gene features was the top performing pipeline with the RF model (held-out test set accuracy = 65.1% [58.3%, 71.9%], brier score = 0.225 [0.208, 0.241], AUROC curve = 0.678 [0.601, 0.756]) (S26 Table). The most important gene feature for the RF model from PVI was from the *AHNAK2* (ENSG00000185567) gene (Table 2, S28 Table, S4 Fig). Model performances for all filtering strategies and datasets for the loss of smell and/or taste phenotype within the AoU cohort is included in the supplemental documentation (S26 Table).

**Table 3:** AoU top feature selection pipeline datasets for the loss of smell and/or taste phenotype held-out test set performance.

CMI-5, LR (chi-squared test), DT-VI_1000_1 (9 features)								
	DT	RF	Lasso	NB	SVM-L	SVM-P	SVM-RB	Elastic Net
Brier Score	0.276	0.328	0.253	0.302	0.253	0.272	0.274	0.253
Accuracy	56.1%	55.0%	57.1%	61.9%	51.9%	54.5%	55.6%	57.1%
AUROC Curve	0.534	0.534	0.571	0.574	0.560	0.524	0.563	0.571
CMI-5, LR (F-Test), DT-VI_1000_1 (10 features)								
Brier Score	0.259	0.277	0.255	0.385	0.256	0.256	0.251	0.254
Accuracy	56.1%	59.8%	53.4%	61.4%	57.7%	57.1%	57.1%	53.4%
AUROC Curve	0.510	0.544	0.554	0.549	0.540	0.536	0.522	0.554
CMI-5, LR (chi-squared test), DT-VI_1000_1 + CMI-5, LR (F-Test), DT-VI_1000_1 (19 Features)								
Brier Score	0.276	0.271	0.258	0.381	0.257	0.275	0.270	0.256
Accuracy	56.1%	57.1%	57.1%	61.9%	57.7%	57.7%	58.2%	57.7%
AUROC Curve	0.534	0.554	0.592	0.591	0.588	0.556	0.552	0.592
CMI-5, LR (chi-squared test), DT-VI_1000_1 + CMI-5, LR (F-Test), DT-VI_1000_1 with Covariates								
Brier Score	0.243	0.222	0.232	0.380	0.231	0.231	0.222	0.229
Accuracy	61.9%	65.6%	68.8%	61.9%	67.2%	67.7%	68.3%	68.8%
AUROC Curve	0.554	0.677	0.671	0.655	0.668	0.664	0.687	0.671
Covariates Only								
Brier Score	0.223	0.289	0.213	0.218	0.212	0.212	0.211	0.212
Accuracy	61.9%	59.8%	61.4%	62.4%	61.4%	61.4%	61.4%	61.4%
AUROC Curve	0.649	0.593	0.708	0.710	0.708	0.707	0.708	0.710

### Testing All of Us cohort models on the University of Iowa cohort

In turn, we extracted the top performing AoU cohort variant dataset features from the UI cohort to validate on an independent dataset. We match the phenotype of loss of smell and/or taste for both cohorts. The validation results were similar to the results of the validation in the reverse direction, with DT (brier score = 0.266), NB (AUROC curve = 0.570), and support vector machine with radial basis kernel function (SVM-RB) (accuracy = 53.3%) models having the strongest held-out test set performance (S29 Table). Comparing the AoU top variant dataset with the top datasets from the UI cohort analysis, there were no intersection of features or variant positions between the datasets. There was an intersection of features associated with the same genes, with the AoU dataset intersecting the *EGFL7*, *FLRT1* (ENSG00000126500), and *OAS*3 (ENSG00000111331) genes with the UI loss of taste variant dataset and intersecting the *LILRB1* (ENSG00000104972) gene with the UI loss of smell dataset (S22 Table).

### Replication analysis of previously identified variants in UI and AoU cohorts

We tested the 28 variants previously found to be significantly associated with the loss of smell or taste symptoms with COVID-19 for association in the UI cohort and the AoU cohort [13]. In the UI cohort, 27 of the 28 variants were present and with all statistical association test performed, none of the variants or variant features were significantly associated with the loss of smell and/or taste phenotype response variable (S30 Table). Within the AoU cohort, no variants or variant features were significantly associated with the loss of smell and/or taste phenotype (S30 Table).

### Pathway enrichment analysis of top performing feature sets

The top performing datasets for the variant and gene ML analyses for the UI cohort and AoU cohort were examined with Ingenuity Pathway Analysis (IPA). Individual dataset analysis resulted with 3 datasets with significant overlap of pathways, the UI loss of taste variant analysis dataset, the UI loss of taste and/smell gene analysis dataset, and AoU gene analysis dataset with 72, 2, and 8 significant pathways, respectively (S31 Table). We found trends within the comparison analysis results showing top overlapping enriched pathways involved in similar biological processes such as immune response, neuronal signaling, and calcium signaling (S32 Table). The majority of the top pathways have overlap with at least 6 of the 8 datasets (S32 Table). The upstream regulator analysis found 3 molecules overlapping at least four of the datasets including both cohorts (S33 Table). Canonical pathway hierarchical clustering resulted in the AoU variant analysis dataset and the UI loss of smell and/or taste variant analysis dataset to be clustered, being the most similar, followed by UI loss of smell variant analysis dataset similar to both datasets. This demonstrates similarities across cohorts with the top performing datasets for each cohort.

## Discussion

In this study, we investigated the host genetic variants and genes associated with anosmia and ageusia symptoms of COVID-19 in two separate cohorts with multiple feature selection strategies and ML methods. We successfully developed novel feature selection pipelines for variant and gene analysis using machine learning models. These models accurately classified patients with and without anosmia and/or ageusia symptoms, significantly outperforming a majority class model. To our knowledge, this is the first study to utilize whole genome sequencing data and ML techniques for gene and variant candidate prioritization and association with anosmia and ageusia with COVID-19. This study improves our understanding of SARS-CoV-2 infections and COVID-19, and specifically the anosmia and ageusia symptoms that impact the daily lives of individuals afflicted by them.

Our genome sequencing analysis included rare and common variants in a joint analysis with the rationale that all variants have an impact on overall biological mechanisms. Including variants with higher and lower allele frequencies in the population would provide a more comprehensive model and analysis. Several studies have used ML for rare variants association with diseases and conditions such as schizophrenia and ovarian failure [29–32]. Symptom development and severity of COVID-19 has been shown to have a genetic component [33–35]. The genetics underlying symptoms such as anosmia and ageusia in COVID-19 may act as genetic modifiers, with an individual's genetic background influencing their development with an infection. COVID-19 symptoms are also considered to be complex genetic traits [36]. Both common and rare variants have been documented as genetic modifiers and to be involved in the development of complex disease traits [37–41]. A previous GWAS assessing common polymorphisms identified 28 variants associated with COVID-19-related loss of smell or taste. However, these variants were not validated in a separate set, and we did not find association of the variants in UI or AoU cohorts, nor was a polygenic risk score evaluation performed. [13]. Although our cohorts were underpowered for a GWAS, other studies have shown significantly informative results with smaller sample sizes using genomic data analyzed with ML methods [23,42]. Our goal was to utilize dataset agnostic feature selection strategies for non-bias selection of the most informative features and perform a ML analysis. This allowed us to gain insight into the genetic etiology of anosmia and ageusia with COVID-19 and establish an optimized and robust workflow for the potential analysis of other diseases.

Each cohort included cases of individuals reporting loss of smell and/or taste within the estimated population prevalence for western countries [6], potentially allowing for more generalizable features to be selected and models to be trained. Optimization of the feature selection pipeline was carried out using the UI cohort with the anosmia phenotype. This approach is responsible for the anosmia variant and gene analyses performing best overall. Validation analysis for both cohorts revealed that the top-performing features and models had poor performance and lacked generalizability to samples in the opposite cohort. Plotting the principal components for each cohort together showed more variation due to the differences in the cohorts rather than differences in the cases and controls, explaining the poor validation performance and the inability of one dataset to inform predictions in the other. These results potentially suggest a more complex genetic basis within the population for anosmia and ageusia symptoms with COVID-19. The AoU cohort results may provide insight into the overall complexity of genes and variants associated, while the UI cohort, with its more limited sample selection, was more focused and found a subset of genetic patterns. Differences with the correlated covariates sex and age also demonstrates the variance between cohorts. A lack of association with the 28 variants previously found to be associated with loss of smell or taste with COVID-19 through GWAS [13] supports the notion of high degree of genetic complexity. All models within each cohort, and for each phenotype in the UI cohort analysis, underwent testing on a complete held-out test set, achieving a local validation. Although the same features, variant or gene, did not allow for validation across cohorts, the overlap of gene functions implicated in the analyses along with several of the same enriched pathways demonstrated both cohort analyses were targeting and finding patterns in the same biological processes.

The top candidate genes from the UI cohort investigating the anosmia phenotype was *SLC2A11* and *SNX29P2*. GLUT11 (*SLC2A11*) has a function of transporting glucose and fructose across the cell membrane and has a low to moderate baseline level of expression in most cell types including cells obtained from the nasal cavity and immune cells [43–45]. GLUT11 involvement aligns with a supported hypothesis for the loss of smell involving the decrease in glucose and lack of energy for olfactory cilia [9]. The cases/control variant allele counts trends with a decrease in function hypothesis of GLUT11, however, rs4428104 is intron variant with unknown significance. Intron variants may be in an unknown functional element directly influencing gene function or regulation, or they could be in linkage disequilibrium (LD) with the causal variant(s). *SNX29P2* product is hypothesized as a modulator of Sorting Nexin 29 (*SNX29*) with little known about either gene [46], although SNX29P2 may have a function in immune cells [47].

The top candidate genes from the UI cohort investigating the ageusia phenotype was *AKR1C2*, *GRIN3A*, and Y RNAs. *AKR1C2* encodes an aldo-keto reductase reacting with substrates such as bile, steroids like testosterone, and neurosteroids [48,49]. Expression of *AKR1C2* has been found in human tongue tissue and there is evidence of AKR1C2 protein interaction with the SARS-CoV-2 M protein [50–52]. There could potentially be a role involving competition of AKR1C2, an unknown substrate, and the SARS-CoV-2 virus in tissue involving taste reception, or with systemic upregulation of SARS-CoV-2 cellular entry factors such as transmembrane serine protease 2 (TMPRSS2) [53]. GRIN3A is a subunit of the N-methyl-D-aspartate (NMDA) receptors and a member of the superfamily of glutamate-regulated ion channels. *GRIN3A* is involved in glutamate neuronal signaling with associated pathways intersecting across cohort and phenotype analyses in the present study. Glutamate has been shown to be the neurotransmitter of olfactory and gustatory signaling and GRIN3A protein function could potentially be involved with the anosmia and/or ageusia symptoms with COVID-19 [54,55]. Y RNAs are conserved non-coding RNAs that appear to have a function with chromosomal DNA replication [56]. Certain Y RNAs have been found to be associated with severity of symptoms with a viral infection, including COVID-19 [57].

The top candidate genes from the UI cohort investigating the anosmia and/or ageusia phenotype were *TRIM68*, *PDGFB*, and *GNAO1*. The TRIM68 protein functions as E3 ubiquitin ligases and has known functions inside inflammatory pathways. TRIM68 decreases or stops production of interferon with viral detection [58]. The missense variant (rs2231975, C442Y) could potentially alter the structure or function of TRIM68 protein in a way that is activated in the presence of a SARS-CoV-2 infection to contribute to or protect from the development of loss of smell and/or taste. Given the amino acid position and TRIM68 protein structure, the loss of cysteine likely would not affect disulfide bonding, while the change to tyrosine may enable post-translational phosphorylation of the tyrosine side chain hydroxyl group. The protein encoded by *PDGFB* plays a role in a wide range of processes including angiogenesis [59]. A previous study has implicated *PDGFB* with susceptibility and severity of COVID-19 symptoms [60] and it has been hypothesized that *PDGFB* could be involved with an enhanced immune response with a SARS-CoV-2 infection [61]. The case/control alternative allele counts for the associated variants with *TRIM68* (rs2231975) and *PDGFB* (rs17303681) show higher counts in controls than cases. For *TRIM68*, this trend could suggest a protective effect of the variant and for *PDGFB* the trend aligns with a damaging effect, but in this scenario, no development of symptoms. *GNAO1* encodes the alpha subunit of the Go heterotrimeric G-protein signal-transducing complex and has been associated with neurodevelopmental disorders [62]. The GNAO1 protein is also involved in the release of inflammatory mediators from immune cells [63] and has been associated with inhibitory signaling in olfactory transduction in rats [64].

The top candidate genes from the AoU cohort investigating the anosmia and/or ageusia phenotype were *EGFL7*, *GRM1*, and *AHNAK2*. *EGFL7* encodes an endothelial cell secreted protein that plays a role in vasculogenesis, angiogenesis [65], and is involved in calcium binding [66]. In recent studies, EGFL7 was implicated in COVID‐19 severity [67] and *EGFL7*-knockout mice displayed dysfunction in olfactory behavior [68]. The case/control alternative allele counts for the associated variant (rs2297538, V153I) trends with a decrease in proper function hypothesis of EGFL7. The substitution of a valine to isoleucine is a chemically conservative variant with a solvent-exposed side chain, likely not disrupting the protein structure alone. However, the loss of the valine could potentially affect protein binding or domain-specific functions or interactions. The *GRM1* gene encodes a metabotropic glutamate receptor that is expressed in taste buds and is involved in umami taste perception and included in the canonical pathway sensory perception of taste [69]. The *AHNAK2* gene encodes a nucleoprotein that is associated with calcium channel proteins [70]. In a previous study, a rare variant analysis investigating genetic association with severe COVID-19 identified a strong signal from a variant in the AHNAK2 gene [71].

**Fig 4:**
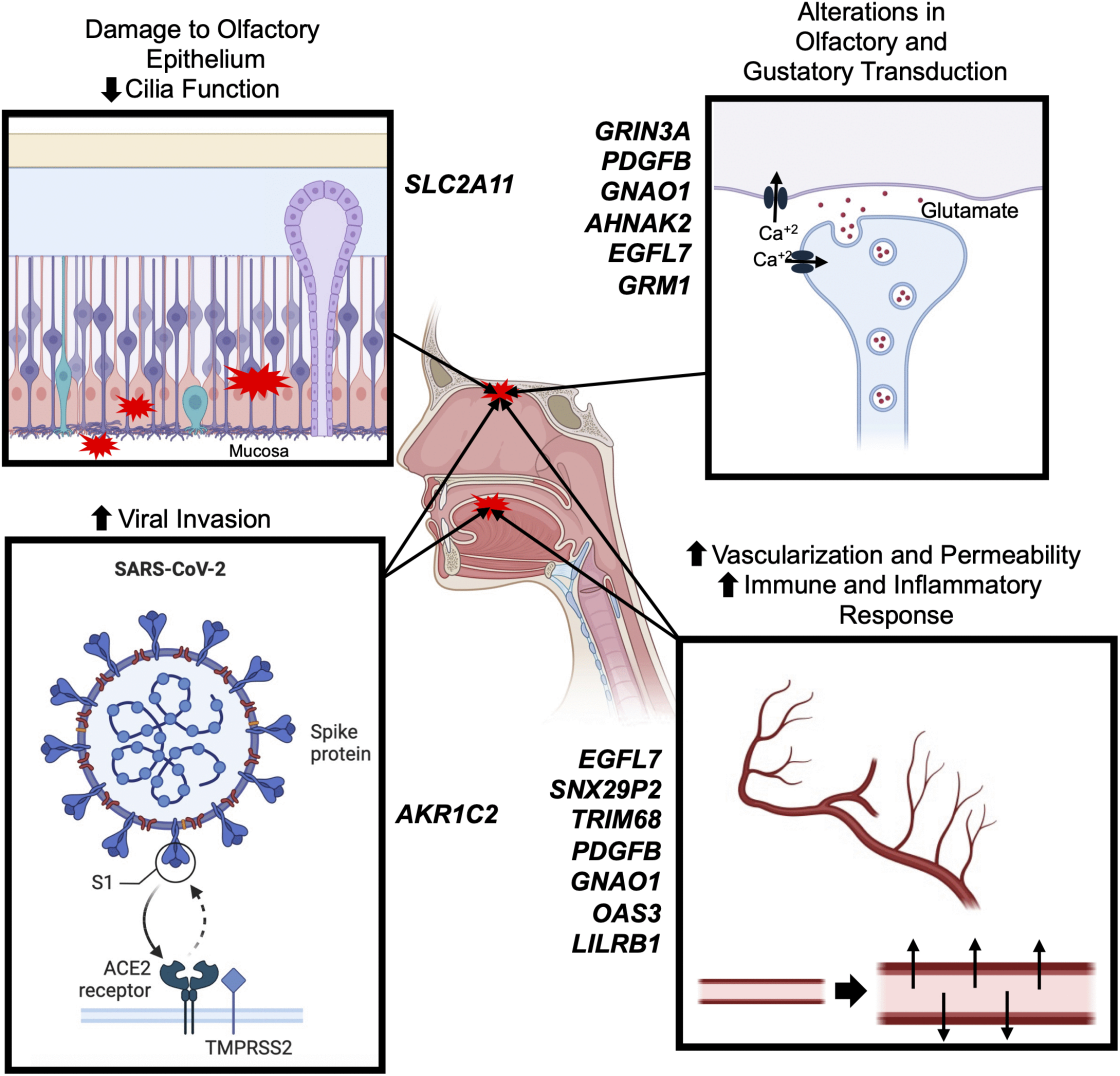
Summary of hypothesis for the involvement of prioritized candidate genes and biological processes in the development of anosmia and ageusia with COVID-19. Created in BioRender. Pietan, L. (2025) https://BioRender.com/h04s965.

By examining the trends within and across the datasets, phenotypes, and cohorts we can obtain a more general view of host genetics involved with the loss of smell and taste in COVID-19. We had several features, genomic positions, and genes intersect between datasets and phenotypes within the UI cohort, and four genes, *EGFL7*, *FLRT1*, *OAS3*, and *LILRB1* overlap between the two cohorts. *OAS3* and *LILRB1* are both involved in immune response [72,73] and *OAS3* has been implicated in COVID-19 susceptibility and severity [74]. The IPA comparison analysis of all top performing feature sets highlighted three biological processes, immune response, neuronal signaling, and calcium signaling, that are in line with the implicated genes described above. Overall, the results of this study provide additional evidence for previous hypotheses proposed for the loss of smell and/or taste with COVID-19 [7–11]. Our results provide support for the potential compound involvement of multiple processes. Starting with the increase in vascularization and/or decrease in vascular integrity (*EGFL7*) to the olfactory epithelium and gustatory nerves, allowing for an increased immune and inflammatory response assisted by other factors such as *SNX29P2*, *TRIM68*, *PDGFB*, *GNAO1*, *OAS3,* and *LILRB1*. The increased inflammation potentially causes damage to the olfactory epithelium, olfactory receptor neurons, and gustatory nerves, and is accompanied by an increase in viral invasion (*AKR1C2*) of gustatory nerves and the olfactory epithelium including the support cells. Damage of the support cells alters the olfactory epithelium mucus contributing to the decrease in energy for olfactory cilia function (*SLC2A11*). Altogether with the potential alterations in olfactory and gustatory transduction with factors such as *GRIN3A* and *GRM1* glutamate neuronal signaling, *GNAO1* involved signaling, and calcium signaling (*GRIN3A, PDGFB*, *GNAO1, AHNAK2, EGFL7*, *GRM1*). These processes, altogether or in subsets, may cause the loss of smell and/or taste with COVID-19 (Fig 4).

A main strength of this study is the robust ML pipeline and analysis. With whole genome sequencing data, we examined numerous feature selection strategies to cut down on the high dimensional nature of the data in a performance enhancing way. We chose not to introduce dataset specific biological filters to increase our chances for novel findings. Our established pipelines were optimized on the UI anosmia phenotype dataset. When validating on other datasets, variations of the pipeline were able to achieve insightful performance demonstrating that these feature selection algorithms, individually and in combination, can provide informative results with ML models. The variations of the pipeline should be tested with new datasets in search of optimal performance. In line with best practices, we implemented the 80/20 dataset split, to create a training/validation set and a held-out test set. The split was performed before feature selection, eliminating any biases and data leakage. Hyperparameter tuning of the ML models was implemented inside a stratified 10-fold CV, altogether estimating performance on validation sets and assessing true predictive performance on a held-out test set within cohorts. Model selection with 10-fold CV was based on the brier score. Model selection based on other metrics yielded weaker results when assessed on the held-out test set. While assessing overall pipeline and model performance, both training and test set performance were considered, with priority given to the brier score, accuracy, and AUROC curve metrics. Multiple well-established and validated ML models were tested and had previously been demonstrated to produce insightful results on biological datasets [14]. With our whole genome sequencing top performing datasets and pipelines, tree-based models, SVM models, Lasso, and Elastic Net were consistently at the top for test set performance and demonstrated less overfitting. With all datasets and pipelines tested for the UI anosmia phenotype, the SVM-RB model demonstrated slightly less consistency, notably with gene analysis pipelines. The DT model does have weaker performance in general but when paired with a DT-VI filter it had a more notable performance with several datasets. Lasso and Elastic Net have the advantage of being interpretable as logistic regression models and DT models can be visualized for interpretation. To conclude the pipeline, PVI assessment for each model was completed and is an important element for the overall goal of the study. Resulting PVI metrics can be compared across models and are not model specific. Ultimately, PVI, feature selection, and pathway analysis allowed for genomic factor prioritization.

The present study is not without limitations. One of the main limitations is the sample size of the UI cohort and incomplete validation of results on an independent dataset. With limited samples sizes, the features selected, and models trained may not be generalizable to the overall population and only target a subset of the population. Future studies aim to validate the results obtained from the UI and AoU cohorts in other datasets. Another limitation is with patient self-reporting of their COVID-19 symptoms through surveys with yes/no answers in the UI and AoU cohort. Asking patients in person or through surveys to assess their own symptoms without clear definition (metric-based) or complete understanding of the symptoms, potentially with significant time after symptoms subsided, leads to phenotypic heterogeneity. This could manifest as inconsistencies between cases and controls, as symptom awareness and interpretation can be influenced by an individual's condition and background, as well as their understanding of the intricacies of the anosmia and ageusia symptoms. This includes distinctions such as transient anosmia associated with mucosal edema versus persistent anosmia, and the understanding of true loss of taste versus loss of flavor due to olfactory dysfunction. The phenotypic heterogeneity adds noise to the machine learning analysis, dampening and/or biasing the genetic associations and the conclusions drawn. The binary phenotype derived from the yes/no survey responses captures a broad definition of anosmia/ageusia with COVID-19, whereas more granular, clearly defined, and metric-based phenotyping would likely yield more precise genetic associations and better resolve distinct pathophysiological mechanisms underlying these symptoms. Future studies plan to incorporate deep phenotyping and metric-based chemosensory testing to better define these phenotypes, address these limitations, and improve the resolution of our genetic association analyses. The final main limitation with the analysis is not having the ability to control for SARS-CoV-2 infection variant/strain or including a temporal and geographical (AoU cohort) assessment of cases and controls. The omicron SARS-CoV-2 variant was first reported November 25^th^, 2021 and presents with lower rates of anosmia/ageusia symptoms than earlier SARS-CoV-2 variant infections [75–77]. Using data collection (UI cohort) and survey entry (AoU cohort) dates as proxies, all participants were infected with a SARS-CoV-2 variant that preceded omicron and exhibited similar rates of anosmia/ageusia symptoms. For future studies we plan to further validate and assess our variant and gene ML pipeline analysis on additional disease datasets.

In conclusion, we sought to prioritize candidate variants and genes involved in the development of anosmia and ageusia with COVID-19 using a novel pipeline of feature selection techniques, ML methods, and a variable importance assessment. We examined two separate cohorts and prioritized variants and genes in overlapping biological processes including immune response, neuronal signaling, and calcium signaling. These findings align with existing literature and offer additional support to previously proposed hypothesis in the field for the development of anosmia and/or ageusia with COVID-19.

## Materials and methods

### University of Iowa COVID-19 whole genome sequencing cohort (UI cohort)

Patients were recruited from the 30^th^ of June 2020 to the 31^st^ of July 2022, and the participant or their lawful authorized representative (parents or guardians for minors) signed an informed written consent following approval from the University of Iowa Institutional Review Board (University of Iowa IRB protocol # 202005052). This study was conducted in accordance with the Declaration of Helsinki and Belmont Report. In total, 187 patients participated, all reporting a positive SARS-CoV-2 infection with antibody or polymerase chain reaction (PCR) testing performed at the University of Iowa Hospital and Clinics and surrounding areas or presumed positive by being symptomatic and in a household with someone who tested positive. Eighteen symptoms were collected from patients through surveys or their electronic medical records including fever, chills, dry cough, productive cough, productive cough with blood, fatigue, muscle or joint pain, sore throat, nausea or vomiting, loss of sense of taste (aguesia), loss of sense of smell (anosmia), rash, runny nose or congestion, headache, shortness of breath, cold sores or sores in the mouth, diarrhea, conjunctival congestion, and no symptoms. The anosmia and/or ageusia phenotypes were constructed from this data and all symptoms were used to assess dataset consistency with population statistics for COVID-19. Loss of smell, loss of taste, and a combined loss of smell and/or loss of taste are the phenotypes that were assessed in the UI cohort with the analysis described below. Patients with COVID-19 and reporting either a loss of smell and/or a loss of taste were labeled as cases and patients with COVID-19 and did not have a loss of smell and/or loss of taste were considered controls. All patients included in the analysis had no documented history of olfactory or gustatory dysfunction prior to COVID-19 diagnosis. Patients under four years of age at time of consent were excluded from the study due to possible inability to assess and convey symptoms. All participants were recruited and samples collected before December 2021.

Whole blood or saliva was obtained from each patient for DNA extraction and whole genome sequencing. Saliva samples were collected in OGR-600 collection kits (adults) or OC175 collection kits (minors too young to spit, or adults with trouble spitting) (DNA Genotek, Ontario, CAN). Symptom surveys were returned with saliva samples. DNA was extracted using PrepIT reagent and ethanol precipitation per manufacturer's instructions (DNA Genotek). Blood samples were collected in EDTA tubes from consented patients. Symptom surveys were collected after consent. DNA was extracted using Gentra Puregene Blood Core Kit C according to manufacturer's instructions (Qiagen, Hilden, Germany). DNA was assessed for purity on a Nanodrop 2000 (260/280 >= 1.8; 260/230 >= 1.0) (Thermo Fisher Scientific, Waltham, Massachusetts, USA). DNA concentration was measured by fluorescence (Qubit 2.0, BR assay), DNA was diluted, checked for fragmentation with 1% agarose gel, and concentration confirmed by HS Qubit assay (Thermo Fisher Scientific). DNA was submitted for whole genome sequencing to Novogene (Sacramento, California, USA) or the Genomics Division of the Iowa Institute of Human Genetics and sequenced using a NovaSeq6000 (Illumina, San Diego, California, USA).

Dataset curation, preprocessing, and machine learning analysis was performed on the University of Iowa’s high-performance computing cluster. Short read whole genome sequencing data was processed using the DRAGEN Bio-IT Platform V3.8 from Illumina. The DRAGEN DNA Pipeline (DRAGEN Host Software Version 07.021.602.3.8.4 and Bio-IT Processor Version 0x18101306) mapped reads to the GRCh37 reference genome and variants were called with default parameters. Samples included in the UI cohort had a mean coverage depth of 32.8X. Sequencing statistics were calculated using MultiQC [78]. Individual samples were jointly call and quality control filtering of variants was performed with the Genome Analysis Toolkit (GATK, version 4.1.5.0) using the following thresholds, DP < 8.0 (Depth), QD < 2.0 (QualByDepth), QUAL < 30.0 (Quality), SOR > 3.0 (StrandOddsRatio, FS > 60.0 (FisherStrand), MQ < 40.0 (RMSMappingQuality), MQRankSum < −12.5 (MappingQualityRankSumTest), ReadPosRankSum < −8.0 (ReadPosRankSumTest) [79]. The Ensembl Variant Effect predictor (VEP, version 105) was used to annotate variants for gene symbols and Combined Annotation Dependent Depletion (CADD) scores with the pick_allele option set to only annotate one block of consequence data per variant allele [80,81]. Samples were further assessed for outliers with PLINK v1.9 [82]. Sex, age, and age squared were examined as potential covariates to include in models. Chi-squared test and logistic regression was performed to determine any association of covariates with the response variable. No ancestral correction was performed as the majority of individuals included in the study were white, non-Hispanic. However, to confirm no correction for residual population structure was needed, PLINK v1.9 logistic regression association tests with different number of principal components (PCs) were used and visualized with quantile–quantile plot (Q-Q plot). The PCs and results conforming most to ideal and bias controlled conditions were used in subsequent analyses. For the machine learning analysis, additional filtering was performed to exclude variants with multiple alternative alleles and include only variants within coding regions of the genome as annotated by GENCODE v42 with 20 base pairs at each end. The bed file was obtained using the UCSC Table Browser and filtering was accomplished using BCFtools (version 1.12) [83–85]. No minor allele frequency filtering was performed as to include rare and common variants in the genomic feature association analysis.

### All of Us Research Program COVID-19 whole genome sequencing cohort (AoU cohort)

The All of Us Research Program Controlled Tier de-identified participant data was obtained and analyzed through the Researcher Workbench and accessed on the 25^th^ of January 2024 [86]. All analyses for the AoU cohort were performed on the Cloud Analysis Terminal powered by the Google Cloud Computing Platform. Through the Cohort Builder, individuals were selected based on availability of short read whole genome sequencing data and their participation and answers in the COVID-19 Participant Experience (COPE) Survey. Participants were included in the present study if they answered “Yes” to the question “Were you tested for COVID-19 in the past month?, “Yes” to the question “Was the test for COVID-19 positive?”, and reported their symptoms to the question “Which of the following symptoms did you have? (select all that apply).”, did not skip, and had no previous loss of smell or taste perception. The symptom options available were, “A fever/feverish,” “Chills or shivers (feeling too cold),” “Unusual fatigue,” “Unusually strong muscle pains/aches,” “Skipping meals,” “Cough,” “Sore or painful throat,” “Difficulty breathing or shortness of breath,” “Unusually hoarse voice,” “Unusual chest pain or tightness in your chest,” “Runny or stuffy nose,” “Loss of smell or taste,” “Unusual eye soreness or discomfort (e.g., light sensitivity or excessive tears),” “Raised, red, itchy, welts on the skin or sudden swelling of the face or lips,” “Headache,” “Dizziness or light-headedness,” “Confusion, disorientation, or drowsiness,” “Unusual abdominal pain or stomach ache,” “Diarrhea,” “Nausea, “Red/purple sores or blisters on your feet, including your toes,” and “None of the above (if selected, no other response options are available.” Only the anosmia and/or ageusia phenotype was available and assessed in the AoU cohort. Participant case/control classification for the anosmia and/or ageusia phenotype was determined by the inclusion or absence of “Loss of smell or taste” in the symptoms reported. All versions of the survey were used, and questions were required to be answered within the same survey attempt based on the survey_datetime. Multiple attempts of the survey by participants were screened to ensure symptoms were consistent across attempts. All participants had survey data entry dates prior to April 2021. To control for ancestral background within the dataset, the cohort was filtered to only include participants that were classified as European (eur) according to the All of Us genetic predicted ancestry. Samples were filtered if they were included in the flagged samples documentation determined by the All of Us sample QC pipeline. In total, the cohort consisted of 947 samples. Sex, age, and age squared were examined as potential covariates to include in the analysis. A chi-squared test and logistic regression was performed to determine any association of covariates with the response variable and included if association was found. Short read whole genome sequencing data was extracted from the database using the described cohort. The AoU cohort samples had a mean coverage depth of 38.8X. Variants were filtered according to the All of Us variant QC pipeline and recommendations. For the machine learning analysis, additional filtering was performed to exclude variants with multiple alternative alleles and include only variants within exon regions of the Gencode v42 basic transcripts provided by the All of Us exome file. Filtering was accomplished using BCFtools (version 1.12) [83,85]. No minor allele frequency filtering was performed as to include rare and common variants in the genomic feature association analysis. Variants were annotated for gene symbols and Combined Annotation Dependent Depletion (CADD) scores with the pick_allele option set to only annotate one block of consequence data per variant allele with VEP (version 105) [80,81].

### Cohort comparison

VCF files from the UI cohort and the AoU cohort were used to perform a principal component analysis (PCA) using PLINK v1.9 to compare the essential features of the two datasets and assess similarity [82]. Individual cohorts were assessed with cases and controls (loss of smell or taste), and several comparisons were made between cohorts with and without case and control annotations. PCA was performed with QC filtered whole genome sets, whole genome sets with intersected variants, variant sets after coding regions and multiple alternative allele filtering (ML dataset), and the intersected variant sets after coding regions and multiple alternative allele filtering. Each comparison was quantified using a permutation test (10,000 permutations), permuting group labels for between group identity-by-state (IBS) differences, where the groups are either cases and controls or cohort designations.

### Machine learning dataset creation

The machine learning analysis was performed in the R (version 4.3.1) and Python (version 3.10.12) programming languages, using R, Python, and Linux command-line libraries, packages, and tools [87,88]. All random seeds were set to seed = 123. The input dataset to the machine learning analysis was created from all variants that passed filtering described above. The python package, VCF Parser, was used to parse the VCF files and create all features (predictor variables) [89]. Four features were generated for each variant across samples: a pair of allele features representing the first and second alleles (encoded as 0 for reference allele and 1 for alternative allele), along with corresponding CADD features with the reference allele encoded as 0 and the alternative allele encoded with the CADD score. The feature names were formatted as chr:pos:gene symbol:feature type, with “chr” and “pos” being the chromosome number and position in the genome of the variant and “feature type” being the first or second allele or CADD feature (e.g., chr9:136670216:EGFL7:Allele2). The target variable or response variable for each dataset was coded as a 1 or 0 for cases and controls, respectively. To address multiple model testing and provide a measure against overfitting and detection of false positive patterns, we performed a random 80/20 split of the full dataset, where 80% of the samples were included in a training set and 20% of the samples reserved for a held-out test set. Features were removed from the training set if 80% or more of the samples recorded a no call for a feature (i.e., “.” recorded for alleles in the VCF file) (NC80 filtering). If the feature is below the 80% threshold, no calls were recorded as 0 for the reference allele. If a feature had zero variance among training samples, it was removed from the training set (ZV filtering). We extensively examined different feature selection strategies and parameter thresholds with variant features and gene features before model training. We selected the top performing strategies based on held-out test set performance metrics of models trained on the filtered features. We tested filters based on mutual information, logistic regression, and model-based features importance. Components of the top performing filtering pipelines are described in the following section. A comprehensive list of filtering strategies and methods tested are described in the supplemental methods (S1 Text).

### Conditional mutual information maximization filtering (CMI)

The input dataset was partitioned into 1000 randomly sampled features without replacement for filtering. The intermediate datasets were input to a conditional mutual information maximization filtering algorithm performed with the Praznik (version 11.0.0) and Recipes (version 1.0.10) R packages [90,91]. This algorithm selects the feature from the dataset with the highest mutual information and selects subsequent features based on the highest information gain given the already selected features. For this study, the number of selected features at each intermediate dataset was 5 features (CMI-5) and 20 features (CMI-20). All features selected from intermediate dataset filtering were included in downstream analysis.

### Logistic regression filtering (LR)

All input features from a dataset are assessed individually with the response variable with logistic regression. P-values were calculated either with a chi-squared test or F-test. All features with p-values exceeding a set threshold are included in downstream analysis. The thresholds used with the top performing pipelines were p-value < 0.10 and p-value < 0.05.

### Decision tree variable importance filtering (DT-VI)

Input datasets were randomly sampled, selecting 1000 features at a time, without replacement, to construct intermediate datasets similar to the conditional mutual information maximization filtering. This method was tested with and without the allele features converted to a factor. Decision tree models were trained on the intermediate datasets for classification of the response variable using the MachineShop R package (version 3.7.0) [92]. Decision Tree model hyperparameter tuning was completed using a grid search with stratified 10-fold cross-validation (CV) for model and parameter selection. Parameter values tested for the decision tree model are listed in supplementary materials (S34 Table). Estimated predictive performance from the 10-fold CV was recorded as mean accuracy. If the best performing decision tree model for an intermediate dataset exceeded the accuracy threshold of a majority class model for the training set, relative permutation-based variable importance (PVI, samples = 25) was performed. All features with a variable importance score > 0 passed the filter and were included in the downstream analysis. Intermediate datasets were either constructed once for filtering, as a filtering step in a feature selection pipeline, or a total of ten iterations were performed with different samplings each iteration. The notation for these algorithms that will be reference throughout are DT-VI_X_Y, with the X being the number of features randomly sampled for intermediate datasets and the Y being the total number of iterations through the algorithm (e.g., DT-VI_1000_1 and DT-VI_1000_10, respectively mentioned in this section).

### Gene feature transformation and analysis

In our gene feature analysis, filtered and non-filtered variant features from the training set were input to a gene feature transformation. The transformation consisted of binning variant features by gene symbol annotation and using an additive approach, summing the features within a bin. All allele features were summed, and all CADD features were summed within a gene, creating two gene features for each gene if features were included in the input dataset. Features not annotated with a gene symbol, were binned in 25 kb windows across the genome, generating region features. The naming convention of these features is gene symbol underscore feature type, with feature type either “Allele” or “CADD” (e.g., SNX29P2_CADD). Three transformations were tested including a no correction transformation, a sample allele frequency correction transformation, and a directional correction transformation. The no correction transformation has no correction and transforms the features as is, directly from the input dataset. The sample frequency correction transformation assesses the frequency of the alternative allele in the training samples. Alternative allele frequencies greater than 0.5 in the training set had the feature encoding swapped, encoding reference alleles as 1 (or their CADD score for CADD features) and alternative alleles as 0. The directional correction transformation assesses each feature and attempts to correct for directional effects. This is done by assessing the ratio of alternative alleles corresponding to cases. If alternative alleles are present more often in controls than in cases or the ratio metric is less than the threshold set at 0.5, the encoding of the feature is swapped. The gene features were then used in downstream analysis either with filtering of the gene features or input to ML model training.

### Feature selection pipelines

Feature (variable) selection (filtering) pipelines will be referenced throughout with the notation X, Y, Z. The X filtering method is performed first with the output of X used for the input to the Y feature selection method, and if applicable, followed by Z. The output dataset of Z is the input dataset to the ML analysis, model training and testing on the held-out test set and PVI analysis. All feature selection pipelines, and the ML analysis were optimized on the UI cohort with the anosmia phenotype. Our initial filtering strategy was CMI-5, LR (p-value threshold = 0.1), DT-VI_1000_1 that we performed across phenotypes and cohorts. Our top performing pipelines were CMI-20, DT-VI_1000_10 for the variant analysis and LR (p-value threshold = 0.05), gene feature transformation (with and without corrections), CMI-20 for the gene analysis, both tested across phenotypes and cohorts.

### Machine learning analysis

After feature selection, models were trained on the training sets. The models assessed were decision tree (DT), random forest (RF), extreme gradient boosted tree (XGBTree), Lasso, Elastic Net, logistic regression (log reg), naïve bayes (NB), support vector machine with radial basis kernel function (SVM-RB), support vector machine with linear kernel function (SVM-L), and support vector machine with polynomial kernel function (SVM-P). The rationale for including this range of models was to evaluate independent association and both linear and non-linear interactions, as different algorithms capture varying types of structure in high-dimensional data. Traditional machine learning methods were specifically prioritized over deep learning approaches due to the sample size, the greater interpretability of classical models for biological feature prioritization, and the increased risk of overfitting and computational overhead associated with neural networks. A grid search with stratified 10-fold cross-validation (CV) was used for hyperparameter tuning and model selection. Hyperparameter values for each model are listed in supplementary materials (S34 Table). 10-fold CV estimate predictive performance was recorded as mean metrics including brier score, accuracy, Cohen’s kappa, area under the receiver operating characteristic (AUROC) curve, sensitivity, and specificity. Models were selected based on brier score, due to brier score being a proper scoring function, with measuring of the accuracy based on prediction probabilities. Held-out test sets were corrected for no calls and features included in model training were selected. For the gene analysis, gene features were constructed in the test sets by selecting the variant features used for the gene transformations in the training set and accounting for any corrected features, if performed. The models selected were used for classification of samples in the held-out test set and true predictive performance of the models were recorded with the same metrics listed above. Multiple performance metrics are recorded to provide a comprehensive evaluation and avoid over-reliance on a single summary statistic. These metrics capture different aspects of predictive behavior and are commonly used across disciplines. We prioritized the brier score, accuracy, and AUROC curve metrics for selecting top-performing pipelines and models and for conciseness. Confidence intervals were calculated at 95% for the top-performing pipelines and models, using the normal approximation method for accuracy, the bootstrap method for brier score, and the DeLong method for the AUROC curve. When evaluating models, training and test set performance was considered to assess across all samples. To further evaluate each model and assess the features that are providing the most important information for predictions, relative permutation-based variable importance (PVI, samples = 25) was calculated and reported for each input feature. The variable importance score is based on the mean change in brier score, and scores are scaled to from 0 to 100, with the most important feature having the score of 100. Model performance was used as a confidence metric for evaluating PVI for prioritizing variant and gene features. Altogether, feature selection, model performance, individually and across models and metrics, and PVI were all used for final candidate variant and gene prioritization.

### Validation

The top-performing datasets and models from the UI cohort were tested on the AoU cohort, and similarly, the top-performing dataset and models from the AoU cohort were tested on the UI cohort. For the UI cohort VCF file, variants present in the final datasets were lifted over to the GRCh38 reference genome using the BCFtools plugin liftover (version 1.18) to construct the datasets for training of models for validation on the AoU dataset and for the validation of the AoU cohort models [93]. For each of the top performing datasets from each cohort, the training and the testing sets were combined, and models were trained on the complete cohort datasets. Variants were matched with reference and alternative alleles across cohorts and variants that were not present in the validation cohort were recorded as 0/0 or the reference allele across all samples. Variants with multiple alternative alleles were encoded as 0 for the reference allele and 1 for all alternative alleles. Models were then tested on the independent held-out test sets and evaluated for predictive performance.

Within each cohort, we tested association of a 28 variant candidate region, found to be significantly associated with the loss of smell or taste phenotype with COVID-19 in a 23andMe cohort [13]. Two sets of features were constructed for the variant set, features with the same encoding strategy as described above for the machine learning analysis with two allele features for each variant and an additive encoding strategy used in the 23andMe cohort genome-wide association study [13]. Three statistical tests were performed to test for association of predictor variables with the response variable, chi-squared test, logistic regression with the Wald test, and logistic regression with a likelihood ratio test. The likelihood ratio test was used to test for association in the 23andMe cohort genome-wide association study [13].

### Ingenuity Pathway Analysis (IPA)

The top performing datasets for the machine learning analysis for both cohorts were submitted for pathway analysis with Ingenuity Pathway Analysis (IPA, QIAGEN Inc.) [94]. We submitted all genes associated with variant or gene features for a core analysis of individual datasets examining overlap with pathways, upstream elements, and disease and biological functions. With the core analysis, default parameters were used and “Anosmia” and “Ageusia” were added as causal networks to be assessed. Analysis significance was determined by uncorrected and Benjamini-Hochberg multiple test corrected p-values. We then performed a comparison analysis of all core analyses performed to assess overlap between datasets, phenotypes, and cohorts with canonical pathways and upstream regulators. Each dataset and pathway are given a score, the negative log of the p-value derived from the right-tailed Fisher’s Exact Test, which we averaged across datasets to yield a rank by overall score.

## Supporting information

S1 Text Description of additional feature selection pipelines examined. (DOCX)

S1 Fig Permutation-based variable importance top 40 features for top performing dataset and models for the UI cohort loss of taste analysis. All features included in the model are assessed. (*A*) Variable importance for the decision tree (DT) model, top performing model for the variant analysis. (*B*) Variable importance for the support vector machine with linear kernel function (SVM-L) model, top performing model for the gene analysis according to the accuracy and AUROC curve metrics. (*C*) Variable importance for the RF model, top performing model for the gene analysis according to brier score.
(DOCX)

S2 Fig Permutation-based variable importance top 40 features for top performing dataset and models for the UI cohort loss of smell and/or taste analysis. All features included in the model are assessed. (*A*) Variable importance for the RF model, top performing model for the variant analysis according to the accuracy and brier score metrics. (*B*) Variable importance for the naïve bayes (NB) model, top performing model for the variant analysis according to the accuracy and AUROC curve metrics. (*C*) Variable importance for the XGBTree model, top performing model for the gene analysis.
(DOCX)

S3 Fig Comparison of UI and AoU cohort datasets cases and controls and cohorts with principal component analysis, plotting the first two principal components. (*A*) UI whole genome sequencing (WGS) dataset. (*B*) UI filtered ML dataset. (*C*) AoU WGS dataset. (*D*) AoU filtered ML dataset. (*E*) UI and AoU filtered ML datasets. (*F*) UI and AoU filtered ML datasets with cases and controls. (*G*) UI and AoU intersection of filtered ML datasets. (*H*) UI and AoU intersection of filtered ML datasets with cases and controls. (*I*) UI and AoU intersection of WGS datasets. (*J*) UI and AoU intersection of WGS datasets with cases and controls. (*K*) UI and AoU WGS datasets. (*L*) UI and AoU WGS datasets with cases and controls.
(DOCX)

S4 Fig Permutation-based variable importance top 40 features for top performing dataset and models for the AoU cohort gene analysis. All features included in the model are assessed. Permutations per feature were performed 25 times and performance is assessed based on mean brier score. Variable importance for the RF model, top performing model for the gene analysis.
(DOCX)

S1 Table Summary statistics for the University of Iowa (UI) cohort. (XLSX)

S2 Table Individual Covariate Association Test with Response Variables. (XLSX)

S3 Table UI cohort loss of smell only phenotype coding and noncoding dataset initial pipeline results. CMI-5, LR, DT-VI_1000_1.
(XLSX)

S4 Table UI cohort loss of smell only phenotype coding regions only dataset initial pipeline results. CMI-5, LR, DT-VI_1000_1.
(XLSX)

S5 Table Complete list of feature selection pipelines examined on the UI anosmia only dataset. (XLSX)

S6 Table Complete 10-fold cross validation training performance and held-out test results for all variant and gene feature selection strategies and pipelines for the UI loss of smell only phenotype. (XLSX)

S7 Table Lists of top datasets and variables from the variant and gene analyses of the UI and AoU cohorts. (XLSX)

S8 Table UI cohort loss of smell only phenotype top variant analysis results. CMI-20, DT-VI_1000_10.
(XLSX)

S9 Table Permutation-based variable importance (PVI) all features for top performing dataset (CMI-20, DT-VI_1000_10) and all models for the UI cohort loss of smell only variant analysis. Permutations per feature were performed 25 times and performance is assessed based on mean brier score.
(XLSX)

S10 Table UI cohort loss of smell only phenotype top gene analysis results. LR (p-value threshold = 0.05), gene feature transformation with the sample allele frequency correction, CMI-20.
(XLSX)

S11 Table Permutation-based variable importance (PVI) all features for top performing dataset (LR (p-value threshold = 0.05), gene feature transformation with the sample allele frequency correction, CMI-20) and all models for the UI cohort loss of smell only gene analysis. Permutations per feature were performed 25 times and performance is assessed based on mean brier score.
(XLSX)

S12 Table UI cohort loss of taste only phenotype top variant analysis results. CMI-5.
(XLSX)

S13 Table Permutation-based variable importance (PVI) all features for top performing dataset (CMI-5) and all models for the UI cohort loss of taste only variant analysis. Permutations per feature were performed 25 times and performance is assessed based on mean brier score.
(XLSX)

S14 Table UI cohort loss of taste only phenotype top gene analysis results. LR (p-value threshold = 0.05, chi-squared), gene variable transformation with the directional correction, CMI-20.
(XLSX)

S15 Table Permutation-based variable importance (PVI) all features for top performing dataset (LR (p-value threshold = 0.05), gene variable transformation with the directional correction, CMI-20) and all models for the UI cohort loss of taste only gene analysis. Permutations per feature were performed 25 times and performance is assessed based on mean brier score.
(XLSX)

S16 Table Complete 10-fold cross validation training performance and held-out test results for all variant and gene feature selection strategies and pipelines for the UI loss of taste only phenotype. (XLSX)

S17 Table UI cohort loss of smell and/or taste phenotype top variant analysis results. CMI-5, LR (p-value threshold = 0.1, F test), DT-VI_1000_1.
(XLSX)

S18 Table Permutation-based variable importance (PVI) all features for top performing dataset (CMI-5, LR (p-value threshold = 0.1, F test), DT-VI_1000_1) and all models for the UI cohort loss of smell and/or taste variant analysis. Permutations per feature were performed 25 times and performance is assessed based on mean brier score.
(XLSX)

S19 Table UI cohort loss of smell and/or taste phenotype top gene analysis results. LR (p-value threshold = 0.05, chi-squared), gene variable transformation with the sample frequency correction, CMI-20.
(XLSX)

S20 Table Permutation-based variable importance (PVI) all features for top performing dataset (LR (p-value threshold = 0.05, chi-squared), gene variable transformation with the sample frequency correction, CMI-20) and all models for the UI cohort loss of smell and/or taste gene analysis. Permutations per feature were performed 25 times and performance is assessed based on mean brier score.
(XLSX)

S21 Table Complete 10-fold cross validation training performance and held-out test results for all variant and gene feature selection strategies and pipelines for the UI loss of smell and/or taste phenotype. (XLSX)

S22 Table Intersection of features, genomic positions, and genes of the top performing datasets from the three phenotype analyses from the UI cohort and AoU cohort. (XLSX)

S23 Table Summary statistics for the All of Us (AoU) cohort. (XLSX)

S24 Table Model performance of top datasets and models from the UI cohort variant and gene analysis tested on the AoU cohort. (XLSX)

S25 Table Permutation test between group identity-by-state differences with plotting PC1 and PC2 from principal component analysis. (XLSX)

S26 Table Complete 10-fold cross validation training performance and held-out test results for all variant and gene feature selection strategies and pipelines for the AoU cohort. (XLSX)

S27 Table Permutation-based variable importance (PVI) all features for top performing dataset (CMI-5, LR (p-value threshold = 0.1, chi-squared), DT-VI_1000_1 + CMI-5, LR (p-value threshold = 0.1, F test), DT-VI_1000_1 with Covariates) and all models for the AoU variant analysis. Permutations per feature were performed 25 times and performance is assessed based on mean brier score.
(XLSX)

S28 Table Permutation-based variable importance (PVI) all features for top performing dataset (LR (p-value threshold = 0.05, chi-squared), gene variable transformation with no correction, CMI-20 with Covariates) and all models for the AoU gene analysis. Permutations per feature were performed 25 times and performance is assessed based on mean brier score.
(XLSX)

S29 Table Model performance of the top dataset and models from the AoU cohort variant analysis tested on the UI cohort. (XLSX)

S30 Table Association test of previously implicated variants for loss of smell and/or taste with COVID-19 in the UI and AoU cohorts. (XLSX)

S31 Table IPA individual dataset Core Canonical Pathway Analysis for top datasets from the UI and AoU cohorts. (XLSX)

S32 Table IPA Comparison Analysis of Core Canonical Pathway for top datasets from the UI and AoU cohorts. (XLSX)

S33 Table IPA Comparison Analysis of Core Upstream Regulators for top datasets from the UI and AoU cohorts. (XLSX)

S34 Table Models and parameter values for hyperparameter tuning. Parameters were tuned with a grid search and 10-fold CV with training sets.
(XLSX)
