# Supplementary material for: Genome-wide machine learning analysis of anosmia and ageusia with COVID-19
Source: PLoS One. 2026 Aug 13;21(8):e0355832. doi: 10.1371/journal.pone.0355832 (PMC13472366; doi:10.1371/journal.pone.0355832)
Supplement: Supplementary file 1 [file pone.0355832.s001.docx]

**S1 Text.** **Description of additional feature selection pipelines examined.**

Best filtering practices were tested on the UI cohort. The order of the filtering methods in the pipeline was determined by initial preliminary results considering the number of features passing the filters and the computation time. DT-VI has a higher computational load than CMI and LR filtering leading to CMI and LR methods to be performed first. Our initial pipeline tested was CMI-5, LR (chi-squared, p-value threshold < 0.1), and DT-VI_1000_1 filtering (majority class accuracy threshold) to confirm this strategy could yield informative machine learning results. Multiple thresholds for CMI and LR methods were tested individually. For CMI, the number of features selected for each intermediate dataset were 5, 10, 20, 100, and 500 features. When applicable, input of the complete dataset was used for CMI filtering selecting 500 features (CMI-Full). For LR, p-values thresholds of 0.1, 0.05, 0.01, 0.005, 0.001, 0.0005, 0.0001, 0.00005, and 0.00001 were tested for chi-squared test and F-test statistics. LR as the initial filtering method was also tested. Testing was performed with model-based variable importance filtering as the initial filter similar to DT-VI_1000_1 with different models. Models tested were DT, RF, Lasso, NB, SVM-L, and XGBTree model with a training set majority class accuracy threshold and variable importance score set to either > 0 or > 75. The RF and Lasso filters were also tested with accuracy thresholds of 60% and variable importance score > 50.

Datasets from the best performing individual filter results and a combination of CMI and LR filtering results were used as input to DT-VI_1000_1 and DT-VI_1000_10 filtering and tested for predictive performance with ML models. DT-VI_X_10 was tested in multiple ways. We assessed the number of features sampled from the initial training set including X = 50, 100, 250, 500, and 1000. We expanded on the DT-VI_50_10 approach by testing accuracy thresholds in two ways, the first by maintaining the same input dataset and testing the accuracy thresholds of majority class (~53%), 60%, 65%, 70%, 75%, and 80%. The second test examined increasing accuracy thresholds in the same manner as the first but using the prior accuracy threshold filtered output dataset as the input dataset to the next test. We expanded on the DT-VI_1000_10 filtering approach by assessing different 10-fold CV estimated performance metrics to select features, with and without allele features converted to factors. The metrics and thresholds assessed were accuracy (thresholds described previously), brier score < 0.25, 0.3, AUROC curve > 0.55, 0.65, sensitivity > 0.5, 0.75, and specificity > 0.5, 0.75. Output datasets from sensitivity and specificity tests were combined to assess the viability of combination effects of features on performance metrics.

The Gene feature analysis tested multiple filtering strategies similar to the variant feature analysis. We used the initial variant feature dataset with NC80 and ZV filtering as the input dataset to the gene feature analysis pipeline. Preliminary analysis examined the performance of transforming the initial training dataset into gene features without any filtering and demonstrated the necessity for filtering before the gene feature transformation. We examined multiple filtering strategies before transformation with both corrections and with no correction including single filters with CMI-20 and LR (p-value threshold < 0.1, 0.05, 0.005) and two filters with all applicable permutations of CMI-20 and LR (p-value threshold < 0.1, 0.05, 0.005) along with DT-VI_1000_1 for the second filter. We expanded the single filter analysis by including a second filter after the gene feature transformation with all permutations of CMI-20 and LR (p-value threshold < 0.1, 0.05, 0.005) for the first filter and CMI-20, CMI-Full, LR (p-value threshold < 0.1, 0.05, 0.01, 0.005, 0.001), DT-VI_1000_1, and DT-VI_50_1 for the gene feature (second) filter. All filtering approaches were assessed by predictive performance of models trained to the data as previously described. A complete list of feature selection pipelines tested is included in S9 Table.
