# Supplementary material for: Genome-wide machine learning analysis of anosmia and ageusia with COVID-19
Source: PLoS One. 2026 Aug 13;21(8):e0355832. doi: 10.1371/journal.pone.0355832 (PMC13472366; doi:10.1371/journal.pone.0355832)
Supplement: Supplementary file 3 [file pone.0355832.s003.docx]

**A**

**B**

**C**

**S2 Fig**. **Permutation-based variable importance top 40 features for top performing dataset and models for the UI cohort loss of smell and/or taste analysis.** All features included in the model are assessed. (*A*) Variable importance for the RF model, top performing model for the variant analysis according to the accuracy and brier score metrics. (*B*) Variable importance for the naïve bayes (NB) model, top performing model for the variant analysis according to the accuracy and AUROC curve metrics. (*C*) Variable importance for the XGBTree model, top performing model for the gene analysis.
