# Supplementary material for: Genome-wide machine learning analysis of anosmia and ageusia with COVID-19
Source: PLoS One. 2026 Aug 13;21(8):e0355832. doi: 10.1371/journal.pone.0355832 (PMC13472366; doi:10.1371/journal.pone.0355832)
Supplement: Supplementary file 4 [file pone.0355832.s004.docx]

**A	B**

**C	D**

**E	F**

**G	H**

**I	J**

**K	L**

**S3 Fig. Comparison of UI and AoU cohort datasets cases and controls and cohorts with principal component analysis, plotting the first two principal components.** (*A*) UI whole genome sequencing (WGS) dataset. (*B*) UI filtered ML dataset. (*C*) AoU WGS dataset. (*D*) AoU filtered ML dataset. (*E*) UI and AoU filtered ML datasets. (*F*) UI and AoU filtered ML datasets with cases and controls. (*G*) UI and AoU intersection of filtered ML datasets. (*H*) UI and AoU intersection of filtered ML datasets with cases and controls. (*I*) UI and AoU intersection of WGS datasets. (*J*) UI and AoU intersection of WGS datasets with cases and controls. (*K*) UI and AoU WGS datasets. (*L*) UI and AoU WGS datasets with cases and controls.
