# Supplementary material for: Genome-wide machine learning analysis of anosmia and ageusia with COVID-19
Source: PLoS One. 2026 Aug 13;21(8):e0355832. doi: 10.1371/journal.pone.0355832 (PMC13472366; doi:10.1371/journal.pone.0355832)
Supplement: Supplementary file 5 [file pone.0355832.s005.docx]

**S4 Fig. Permutation-based variable importance top 40 features for top performing dataset and models for the AoU cohort gene analysis.** All features included in the model are assessed. Permutations per feature were performed 25 times and performance is assessed based on mean brier score. Variable importance for the RF model, top performing model for the gene analysis.
