# Supplementary material for: Genome-wide machine learning analysis of anosmia and ageusia with COVID-19
Source: PLoS One. 2026 Aug 13;21(8):e0355832. doi: 10.1371/journal.pone.0355832 (PMC13472366; doi:10.1371/journal.pone.0355832)
Supplement: Supplementary file 6 [file pone.0355832.s040.docx]

**Comments**

This is an interesting study applying multiple machine learning approaches to identify candidate genes and variants associated with COVID-19–related anosmia and ageusia using two independent cohorts (UI and AoU). The analytical pipeline is now clearly described, and the authors have addressed the concerns raised by previous reviewers. I have one remaining concern regarding patient phenotyping.

Although the authors acknowledge the limitation of symptom-based classification, the potential rate of misclassification inherent in self-reported smell and taste loss deserves more explicit discussion. Subjective chemosensory complaints are influenced by nasal obstruction, mood or cognitive state, and individual awareness, and therefore may not accurately reflect true olfactory or gustatory dysfunction. Importantly, such phenotypic misclassification introduces “noisy labels,” which can directly affect machine learning–based feature selection and variable importance ranking, potentially diluting or biasing genetic associations.

In addition, olfactory and gustatory dysfunction exist along a spectrum from transient to persistent loss, likely reflecting distinct underlying mechanisms. A binary yes/no symptom definition therefore likely captures a broad symptomatic phenotype rather than a strictly sensorineural one. Clarifying this distinction and its implications for model performance and biological interpretation would strengthen the manuscript.

I encourage the authors to address these issues more explicitly in the discussion and to consider how future studies incorporating objective chemosensory testing or more granular phenotyping could improve genetic inference.
