# Supplementary material for: Genome-wide machine learning analysis of anosmia and ageusia with COVID-19
Source: PLoS One. 2026 Aug 13;21(8):e0355832. doi: 10.1371/journal.pone.0355832 (PMC13472366; doi:10.1371/journal.pone.0355832)
Supplement: Supplementary file 7 [file pone.0355832.s041.docx]

Response to Reviewers and Editors

Thank you very much for taking the time to review this manuscript. Please find the detailed responses below and the corresponding revisions/corrections highlighted/in track changes in the revised manuscript files.

Response to Editors Comments

**Comment 1 and 2 on submission format and author contributions:** These comments were address during the online submission of the manuscript.

**Comment 3 and 4 on data availability**:

**Comment 3:** Thank you for stating "The WGS data generated in this study have been submitted to the NCBI BioProject database (https://www.ncbi.nlm.nih.gov/bioproject/) under accession number PRJNA1173221." Please note that, though access restrictions are acceptable now, your entire minimal dataset will need to be made freely accessible if your manuscript is accepted for publication. This policy applies to all data except where public deposition would breach compliance with the protocol approved by your research ethics board. If you are unable to adhere to our open data policy, please kindly revise your statement to explain your reasoning and we will seek the editor's input on an exemption.

**Comment 4:** In the online submission form, you indicated that "Variant data and ML datasets from the UI cohort analyzed during the study are available from the corresponding authors on request." All PLOS journals now require all data underlying the findings described in their manuscript to be freely available to other researchers

**Response**: The WGS data generated in this study and submitted to the NCBI BioProject database (https://www.ncbi.nlm.nih.gov/bioproject/) under accession number PRJNA1173221 will be released once the manuscript is accepted for publication. The BioProject submission PRJNA1173221 of sequencing data contains all data used in the submitted manuscript for the University of Iowa (UI) cohort. All other datasets (for the UI cohort), including variant sets and machine learning (ML) datasets, can be reproduced by accessing the BioProject PRJNA1173221 sequencing data and processing/analyzing the data according to our detailed methods section. The statement "Variant data and ML datasets from the UI cohort analyzed during the study are available from the corresponding authors on request." will be removed during the revised submission to eliminate any confusion as all data is already freely available in a public repository.

Response to Editors Comments after Revision

**Comment 1:** As the reviewer noted, the revision did not adequately address the concerns regarding statistical power (due to the small sample size) and the lack of sufficient biological interpretation.

**Response:** I believe we have now addressed all concerns about biological interpretation with our new revisions. We have also addressed the comments on sample size and believe our methods are reliable and robust to ensure we are detecting true patterns in the data. Our results and conclusions provide insight into the genetic factors involved with anosmia/ageusia symptoms with COVID-19 and will be of interest to scientists in the relevant fields.

Response to Reviewer #1 Comments

**Notes:** In the submitted manuscript, the authors performed genome-wide machine learning (ML) analysis to pinpoint genes/variants accountable for COVID-19 related loss of smell/taste (anosmia and ageusia).

**Comment 1:** One of my big concerns is the limited sample size, with the discovery cohort comprising only 187 sample, while the features are as many as millions. Overfitting is likely to happen in this situation. Also, the validation cohort (N=947) is more than five times larger than the discovery cohort, while the performance is worse. Although the authors tried to explain this comparison using PCA, it seems to me that the explanation is not convincing.

**Response:** Thank you for this comment. We acknowledge that the University of Iowa (UI) cohort sample size is modest and note it as a limitation in the manuscript (Discussion, page 19, lines 484-488). We agree that sample size is a crucial consideration with high-dimensional genomic analyses and when developing/validating machine learning (ML) pipelines. Addressing this concern was central to the study’s design and methodology.

While constrained by sample size, our findings are supported by previous ML-based genomic studies that achieved robust biological insights with similar cohort size and relatively high feature counts (Discussion, page 14, lines 345-347; references 23 and 42). We designed our study to follow ML best practices for limited sample sizes and controlling for bias and overfitting.

To control for overfitting, within each cohort, we implemented a strict training and testing data split strategy: an 80/20 split into training/validation and held-out test sets was performed prior to feature selection, thereby preventing information leakage. This is noted in the Discussion and Methods sections (Discussion, page 18, lines 464-466; Methods, page 24-25, lines 644-653).

We report 10-fold cross validation estimated performance metrics in the supplement to be considered alongside the true predictive performance on the held-out test set for each cohort, feature selection strategy, and model.

We also conducted cross-cohort validation in both directions:

Training on the UI cohort (N=187) and validating on the NIH All of Us (AoU) cohort (N=947), and Training on the AoU cohort and validating on the UI cohort.

While performance was reduced in the AoU cohort analysis (AUROC = 0.687), the models still outperformed a majority class baseline (accuracy = 68.8% vs. 55.5%), indicating that the model and features have discriminative capacity (Results, pages 10, lines 261-265).

Principal component analysis (PCA) is used to control for population stratification or differences in allele frequencies in populations of different ancestry backgrounds in genomic analyses. With the AoU cohort, we selected for individuals with predicted European ancestry to be most similar to the UI cohort (Methods, page 23, lines 604-606). Even with this consideration, the PCA revealed that inter-cohort differences dominate allele variance structure, more than phenotype (case/control) separation demonstrating a lack in ability of one cohort to inform on the other cohort.

We included further clarification in the manuscript (Discussion, page 14, lines 358-360). “*Plotting the principal components for each cohort together showed more variation due to the differences in the cohorts rather than differences in the cases and controls, explaining the poor validation performance and the inability of one dataset to inform predictions in the other.”*

We also provide additional support for cohort differences by demonstrating cohort-specific correlation of covariates (age and sex) (Results, pages 5, lines 117-119; Results, page 10, lines 249-251; Discussion, page 14, lines 364-365). We further support our conclusion of a high degree of genetic complexity with COVID-19-related loss of smell or taste by failing to replicate previously reported GWAS loci (reference 13) and support the viability of each cohort informing on the etiology of the loss of smell or taste with COVID-19 by pathway enrichment results of prioritized gene/variant features highlighting biologically relevant processes that are consistent with previously proposed mechanisms.

**Comment 2:** Another point is that the authors tried a lot of ML methods, but explain little about the results of these methods. They just listed the results there, without reasoning on how to evaluate and combine the results of different ML models.

**Response:** Thank you for this observation. We agree rationale for model selection and our methods for evaluation should be well described. We have made revisions and included the following sentences in the Methods section explaining our rationale for model selection and details on how we are evaluating and interpreting the results of feature selection, model performance, and permutation-based variable importance (PVI).

Methods, pages 27-28, lines 731-736
*“The rationale for including this range of models was to evaluate independent association and both linear and non-linear interactions, as different algorithms capture varying types of structure in high-dimensional data. Traditional machine learning methods were specifically prioritized over deep learning approaches due to the sample size, the greater interpretability of classical models for biological feature prioritization, and the increased risk of overfitting and computational overhead associated with neural networks.”*

Methods, page 28, lines 746-750
*“Multiple performance metrics are recorded to provide a comprehensive evaluation and avoid over-reliance on a single summary statistic. These metrics capture different aspects of predictive behavior and are commonly used across disciplines. We prioritized the brier score, accuracy, and AUROC curve metrics for selecting top-performing pipelines and models and for conciseness.”*

Methods, page 28, lines 752-753
*“When evaluating models, training and test set performance was considered to assess across all samples.”*
Methods, page 28, lines 753-756
*“To further evaluate each model and assess the features that are providing the most important information for predictions, relative permutation-based variable importance (PVI, samples = 25) was calculated and reported for each input feature.”*

Methods, pages 28-29, lines 757-762
*“Model performance was used as a confidence metric for evaluating PVI for prioritizing variant and gene features. Altogether, Feature selection, model performance, individually and across models and metrics, and PVI were all used for final candidate variant and gene prioritization.”*

In terms of combining the results of different machine learning models, our strategy was to assess each pipeline and model individually as well as consider top features of importance across models and noting this in the Results section (page 6, lines 149-153; page 8, lines 196-198; page 8, lines 206-208; page 10-11, lines 265-268). We chose not to combine models into ensembles to preserve interpretability, as our primary goal was to prioritize biologically relevant features to the phenotypes based on their relative importance. In the AoU cohort analysis, we describe our method for combining results of two pipeline with merging two feature set/datasets and retraining models (Results, page 10, lines 255-261).

**Line Comment 1:** Line 128 and line 133 are not clear. What is the difference between preliminary and additional filtering? From line 133, it seems that additional filtering is for non-coding region, so preliminary filtering is for coding region? It is hard to understand the writing.

**Response:** Line 128 (new line 124) mentions “Preliminary testing of our initial filtering pipeline with Conditional Mutual Information-5 (CMI-5), logistic regression (LR), and decision tree variable importance_1000_1 (DT-VI_1000_1) and five ML models…”, with the initial pipeline consisting of CMI-5, LR, and DT-VI_1000_1 filtering. We have made small revisions to be clear on what the pipeline/filtering method is.

*“Preliminary testing of our initial filtering pipeline consisting of Conditional Mutual Information-5 (CMI-5), logistic regression (LR), and decision tree variable importance_1000_1 (DT-VI_1000_1) filtering and five ML models…”*

The interpretation of line 133 (new line 129) with additional filtering consisting of removing variant/features in non-coding regions is correct.

**Line Comment 2:** Line 144. How are the two variants mapped to genes?

**Response:** The two variant features mentioned on line 144 (new line 143) that are in the *SLC2A11* and *LHCGR* genes are mapped by their genomic coordinates (chr and pos) in the VCF file and annotated for gene symbols by Ensembl’s Variant Effect Predictor (VEP) and the Ensembl Transcript Database (Methods, page 22, lines 556-559). Noted variants were also manually confirmed for gene annotation by coordinates in the VCF file.

**Line Comment 3:** Line 178. What is “the top performing feature selection pipelines”? Trained in the loss of smell? If so, why it can be used to investigate the loss of taste and the loss of taste/smell. Lack of an explanation.

**Response:** Thank you for bringing this lack of explanation and clarity to our attention. We have made the following revision to relay our methods and results accurately.

Results, page 8, lines 185-187 *“The top performing feature selection pipelines established during the loss of smell phenotype analysis were used to investigate the loss of taste phenotype and the loss of smell and/or loss of taste phenotype.”*

This consists of 12 feature selection strategies utilized to assess the new phenotypes with results from all pipelines reported in the supplemental documentation (S16 Table; Results, page 8, lines 198-199). The rationale here is that we found a set of feature selection strategies that performed well on the loss of smell phenotype dataset and instead of completing the more extensive exploratory feature selection analysis (255 feature selection pipelines), we only tested the ones that were shown to yield insightful results on a very similar dataset. The full pipelines were performed with the new datasets where the only aspect coming from the loss of smell phenotype analysis is the feature selection strategies. All models for the loss of taste and loss of smell and/or taste phenotypes are trained on their respective training sets.

**Line Comment 4:** Line 644 and 645, in gene feature transformation, it is not accurate if considering only the gene coordinate. The 3D structure of genome is preliminary, only considering the features within a gene bin and summarizing them together may miss important long-distance gene-variant connections.

**Response:** Yes, this is a great point. Thank you for noting this. During our preliminary analysis we tested the inclusion of variants genome wide as well as excluding non-coding regions of the genome (Results, page 5-6, lines 124-135). We found excluding non-coding regions produced higher predictive performance as a base line. Most of long-distance gene-variant connections would most likely be in these excluded regions if they are not included in the GENCODE database, the resource we utilized for extracting the variant in the coding regions. We agree that these regulatory regions are important, but this is a tradeoff in our study design where we optimized to assess potential direct functional impact of variants on proteins and for performance, to potentially provide insight into the genetics involved in the development of the phenotypes. The implementing of the gene feature transformation, will create a 25 kb bin for any variant feature still included in the dataset after non-coding filtering that does not have a gene symbol annotation (VEP, Ensembl transcript database). This could include regulatory regions, and if there is an association with a 25 kb region feature and a gene feature, the ML analysis will still find these patterns and prioritize these features together for the association to be found.

Response to Reviewer #2 Comments

**Notes:** This submission investigates the host genetic basis of COVID-19-associated anosmia and ageusia by integrating whole-genome sequencing (WGS) data from two cohorts (UI and AoU, total N=1,134) with machine learning-driven feature selection. The authors propose a novel variant/gene prioritization pipeline and report candidate genes linked to immune response, neuronal signaling, and calcium signaling pathways. While the work addresses an underexplored aspect of COVID-19 sensory sequelae and leverages methodological innovation in feature selection, several critical issues require clarification to strengthen the validity and impact of the findings.

**Comment 1:** The rationale for prioritizing traditional machine learning over deep learning remains unclear. Given the robust capabilities and mature algorithms of deep learning in classification tasks (e.g., CNNs for sequence data, transformers for high-dimensional feature integration), the authors should explicitly justify their choice of machine learning approaches.

**Response:** Thank you for this comment. We agree, this is a great point that needs to be addressed. The following revisions were made to the manuscript.

Methods, page 27-28, lines 731-736 *“The rationale for including this range of models was to evaluate independent association and both linear and non-linear interactions, as different algorithms capture varying types of structure in high-dimensional data. Traditional machine learning methods were specifically prioritized over deep learning approaches due to the sample size, the greater interpretability of classical models for biological feature prioritization, and the increased risk of overfitting and computational overhead associated with neural networks.”*

**Comment 2:** Similar to the missing heritability phenomenon observed in Genome-Wide Association Studies, the extent of feature selection in the pipeline warrants careful consideration. It is imperative for the authors to demonstrate how model accuracy, AUROC, and other performance metrics vary across different feature selection thresholds.

**Response:** Yes, this is another great point that we took into consideration when designing and implementing our analysis. Our goal was to distill the variant and gene features down in a dataset agnostic manner, where the noise/features that had no biological relation to the phenotype would be removed and all informative and potentially biologically relevant features would be retained. There is a balance here, so that the models are not overwhelmed with noise, but we also do not lose any informative features.

In our analysis we investigate three feature selection algorithms and tested several permutations of these algorithms together across different feature selection parameter thresholds. These methods are described in S1 Text in the supplemental materials and mentioned in the main text (Methods, page 25, lines 661-662). A total of 255 feature selection strategies (S5 Table) were assessed and performance reported in S6 Table. Here we demonstrated how the performance metrics varied across different strategies and parameters. Our strategy involved a grid search–like approach to systematically evaluate the filtering methods and commonly used parameter thresholds individually and in combination. Parameters tested included: p-value cutoffs for GLM (0.1, 0.05, 0.01, 0.005, 0.001, 0.0005, 0.0001, 0.00005, 0.00001), the number of variables selected (CMI: 5, 20; DT-VI: 50, 100, 250, 500), variable importance thresholds for DT-VI (>0, 50, 75), and performance metrics with DV-VI (accuracy, brier score, AUROC, sensitivity and specificity, and at least two threshold values tested). We also tested other model in addition to the decision tree model (DT-VI) for model specific variable importance. Models included were decision tree (DT), random forest (RF), extreme gradient boosted tree (XGBTree), Lasso, naïve bayes (NB), and support vector machine with linear kernel function (SVM-L).

Our Results and Methods sections include the following revision to make this clear.

Results section, page 6, lines 135-138
*“Outside of our initial pipeline to determine the viability of our approach, we extensively examined other feature selection strategies and parameter thresholds. All performance results and trends in performance for all variant and gene feature selection strategies and pipelines are included in the supplemental documentation (S5-S6 Tables).”*

Methods section, page 25, lines 656-658
“We extensively examined different feature selection strategies and parameter thresholds with variant features and gene features before model training.”

**Comment 3:** Sequencing depth directly influences both the number of features and the risk of overfitting, thereby significantly impacting model accuracy and stability. We strongly recommend supplemental analyses evaluating model performance under varying WGS sequencing depths (e.g., subsampling reads or simulating lower-coverage data).

**Response:** Thank you for this analysis suggestion. We agree that sequencing depth can influence variant calling and thereby the number and quality of features used in our analysis. Our study utilizes the University of Iowa cohort dataset and the NIH All of Us dataset that have dataset mean sequencing depths of 32.8X and 38.8X respectively and are consistent with standard best practices quality control metrics for whole genome sequencing data for variant analysis.

Revisions made in manuscript to report sequencing depth statistics.
Methods, page 21, lines 549-550
*“Samples included in the UI cohort had a mean coverage depth of 32.8X.”*
Methods, page 23, line 611-612
*“The AoU cohort samples had a mean coverage depth of 38.8X.”*

Our study focuses specifically on exploring the genetics involved with COVID-19-related anosmia and ageusia. We acknowledge that evaluating method robustness across varying sequencing depths is an important consideration, particularly for generalizing the framework and pipelines methods across studies with differing data quality. However, we believe that such an analysis would be more appropriate and valuable for a methods-focused manuscript which is currently in preparation.

Regarding the number of features and overfitting risk, we note that while increasing depth may introduce additional variants and features, the total feature space is already high-dimensional under standard depth. Therefore, any incremental increase in feature count from even higher coverage/depth is unlikely to alter model complexity in the context of this already large feature set. Furthermore, to control for overfitting, within each cohort, we implemented a strict training and testing data split strategy: an 80/20 split into training/validation and held-out test sets was performed prior to feature selection, thereby preventing information leakage. This is noted in the Discussion and Methods section (Discussion, page 18, lines 464-466; Methods, page 24-25, lines 644-653). These methods help reduce the chance of overfitting the models and increase the chance of obtaining more generalizable insights.

**Comment 4:** The reported AUROCs (0.687–0.798) suggest modest discriminative ability. How does this compare to clinical or demographic predictors alone? Have the authors conducted any statistics and research on this aspect?

**Response:** We did assess two demographic features, sex and age, as covariates in both the UI and AoU cohorts. We found no association in the UI cohort which lead us to not include the covariates in the models (Results, pages 5, lines 117-119; S2 Table; Methods, page 22, lines 559-561). For the AoU cohort, we found an association with the sex and age covariates (Results, page 10, lines 249-251; Methods, page 23, lines 608-610) and included them in the models as well as models without covariates and models with only covariates (Results, page 10-11, lines 254-272; Table 3). With the AoU variant analysis, *“The covariate only models demonstrated slightly stronger performance when considering the metrics brier score and AUROC curve for the held-out test set but weaker performance when considering mean metrics for the 10-fold CV training performance and held-out test set accuracy when compared to the full dataset models”* (Results, page 11, lines 268-271). We did not include any other clinical features as we are most interested in genetics associations with the development of anosmia and ageusia with COVID-19.

**Comment 5:** While the authors have commendably balanced case and control sample sizes in the current study, real-world deployment scenarios often involve substantial class imbalance, particularly when using larger cohorts where control samples may dominate. To ensure robustness and clinical applicability, it is critical to evaluate model performance under imbalanced data conditions.

**Response:** Thank you for this comment. We strongly agree. Within our cohorts and the phenotypes that we investigated we had a balance in our cases and controls with slight variance depending on the analysis. In our study, a balance of cases and controls fits within the real-world prevalence of the loss of smell and taste symptoms with COVID-19 in western countries and is the best study design for robustness and clinical applicability (Introduction, page 2, lines 51-52; Discussion, page 14, lines 352-354; Reference 6). As for generalizability of the methodology for studies that will have an imbalance in case and control samples, this is a great suggestion for an analysis and we will include it in our methods-focused manuscript currently in preparation.

**Comment 6:** Consider adding subheadings to the Results section to improve organizational clarity and reader engagement.

**Response:** Thank you for this suggestion. We have added subheadings to the Results section in the revisions of the manuscript.

Response to Reviewer #3 Comments

**Notes:** The authors employed permutation-based variable importance (PVI) to prioritize candidate genetic variants and genes associated with anosmia and ageusia in COVID-19, leveraging machine learning models on genome sequencing data from the UI and AoU cohorts, achieving AUROC scores up to 0.798 and identifying factors with potential biology.

**Comment 1:** Are these signals related to loss of taste and smell specific to COVID-19 patients? What about non-COVID patients? Would demonstrating this framework on a well-powered dataset yield better results?

**Response:** Thank you for these questions. The genetic signals presented are specific to loss of smell and/or taste in the context of COVID-19, as our study design did not include individuals experiencing anosmia or ageusia unrelated to COVID-19. Therefore, we cannot draw conclusions about the applicability of our findings to non-COVID-related cases of anosmia or ageusia.

Regarding the impact of dataset size on the analysis, we agree that larger, well-powered datasets can improve model stability and potentially increase performance. However, in our analyses of the All of Us cohort, despite being larger than the University of Iowa cohort, yielded weaker model performance when both were individually tested. We interpret this as a reflection of the underlying genetic complexity of the anosmia and ageusia symptoms with COVID-19. Each cohort may capture different facets of this complexity due to differences in population structure, phenotypes, and/or environmental variables.

We believe that the machine learning framework presented here is capable of identifying predictive and biologically informative genetic features when the relevant signal exists within the data. Increased sample sizes and additional data would certainly aid in enhancing the resolution and generalizability of the results, particularly considering the likely polygenic and heterogeneous nature of the phenotypes being assessed here and any other similar study to be performed.

We mention the sample size limitation and discuss briefly points made in this comment response in our discussion (Discussion, page 19, lines 484-488).

**Comment 2:** Permutation-based variable importance may require substantial computational resources. While it may be suitable for this small-scale genetics study, can it be applied to studies with millions of samples?

**Response:** This is a great question, thank you. Permutation-based variable importance (PVI) can be computationally intensive, especially in large-scale datasets. In our study, we used the MachineShop R package, which supports parallel computation of PVI, and executed our analyses on an HPC environment.

We cannot say that our implementation of these methods would be able to handle millions of samples, as we have not yet tested this approach on datasets with millions of samples, or a dataset larger than the AoU cohort, but we note that the underlying framework is scalable in principle. With sufficient computational resources and parallelization, PVI can be applied to larger cohorts keeping in mind the increasing cost in runtime and memory. Given the tradeoff of computational resources and time, other approaches such as GWAS, with millions of samples may be better suited.

For this study, the number of samples and features were well within a range that allowed the application of PVI across multiple models and pipelines, and we found it to be a practical and interpretable method for prioritizing predictive genetic features.

**Comment 3:** What is the potential for this framework to be generalized to other studies?

**Response:** Yes, there is great potential for the framework of the current study to be generalized to other similar studies. We designed our methods and the implementation of the feature selection and ML techniques to be dataset agnostic for the purpose of applying it to multiple studies. We have applied these strategies to other datasets and phenotypes and are developing a pipeline tool for the streamlined use of these methods, but the inclusion of these results is outside the scope of the current manuscript and will be reported in subsequent publications.

**Comment 4:** Some parameters of WGS need clarification, including sequencing depth, MAF filtering, etc.—perhaps I missed them.

**Response:** Thank you for bringing this oversight to our attention. We have made the following revisions to include sequencing depth metrics.

Methods section, page 21, lines 549-550
*“Samples included in the UI cohort had a mean coverage depth of 32.8X.”*
Methods section, page 23, lines 611-612
*“The AoU cohort samples had a mean coverage depth of 38.8X.”*

We did not include any minor allele frequency (MAF) filtering to include rare and common variants in the genomic feature association analysis. Revisions to make this clear are included in the Methods section.

Methods section, page 22, lines 570-571; & page 23-24, lines 616-619
*“No minor allele frequency filtering was performed as to include rare and common variants in the genomic feature association analysis.”*

Response to Reviewer #4 Comments (after revision)

**Reviewer #4 Notes:** This study, by integrating machine learning with genomic sequencing data, has for the first time systematically revealed the host genetic mechanisms underlying olfactory/gustatory dysfunction in COVID-19 patients, identifying significant associations between HLA region variants and calcium ion signaling pathway genes (e.g., CACNA1C). The predictive model constructed using feature selection achieved an AUROC of 0.798 in an independent cohort, validating the effectiveness of the gene prioritization strategy. This paper has strong clinical significance.

**Comment 1:** However, as other reviewers have pointed out, the study faces a sample size limitation, with the UI cohort comprising only 187 cases (79 positive samples), which may affect statistical power and make the results less convincing. Additionally, the AoU cohort consists of 82% white individuals, limiting the generalizability of the results.

**Response:** Thank you for your comments and questions. Within this study, our analysis utilized two independent cohorts, the UI cohort (N = 187) and the AoU cohort (N = 947). We agree the UI cohort sample size is a limitation in this study and noted this in the discussion (Discussion, page 19, lines 484-488). With the UI cohort, we examined 3 different phenotypes (loss of smell, loss of taste, loss of smell and/or taste) all having >95 positive samples, consistent with COVID-19 symptom prevalence seen in the United States population (Introduction, page 2, lines 51-52; Discussion, page 14, lines 352-354; Reference 6). With our analysis, we employed machine learning best practices by utilizing and reporting held-out test set prediction performance metrics with 95% confidence intervals for both cohorts and for the main results to ensure robust model evaluation. This is the best assessment for how well a model is finding true patterns related to the response variable (phenotype) in the data and provides a direct measure of reliability. Most of our dataset and model performance results within cohorts exceeded results from a model training on random patterns supporting the strength of the findings despite modest UI cohort size. We interpret the results, datasets, and features highlighted by the permutation-based variable importance assessment from each of the cohorts and analyses to be pieces of the genetic puzzle or the genetic factors involved with developing anosmia and/or ageusia with COVID-19.

For the AoU cohort, we restricted analyses to individuals of predicted European ancestry to minimize confounding factors due to population stratification (Methods, page 23, lines 604-606). While we agree this limits generalizability beyond European ancestry populations, this approach aligns with standard practices in genomic association studies. Additional rationale for this method is that the anosmia and ageusia symptoms with COVID-19 have been reported at higher frequencies in western populations and having limited qualifying non-European sample sizes in the All of Us data.

**Comment 2:** The biological plausibility of the candidate genes requires multi-dimensional validation (e.g., spatial expression validation using single-cell sequencing data and cross-validation with existing GWAS results).

**Response:** This is a great point, and we agree that additional validation is necessary to confirm the involvement of these candidate genes in the development of anosmia and/or ageusia symptoms with COVID-19. Within our discussion, we performed a literature search for the top candidate genes and noted function and expression in relevant tissue types found by previous studies. An additional spatial expression validation using a single-cell sequencing analysis is out of the scope of the current study. The largest GWAS to be performed for the loss of smell and/or taste symptoms with COVID-19 to date is Shelton et al. 2022 using 23andMe data and is referenced throughout our manuscript. To cross-validate or to incorporate the data into our analysis, we made multiple attempts to obtain the data utilized in Shelton et al. 2022 or to obtain their per variant GWAS summary statistics but were not successful. These attempts were made during the time just prior to 23andMe’s bankruptcy and we had no response from the company.

**Comment** **3:** Did the study adjust for clinical confounding factors such as nasal history and medication use?

**Response:** Yes, all individuals included in the study from both the UI cohort and the AoU cohort had no prior loss of smell or loss of taste reported. This was an oversight not reported in the manuscript and is now included as a revision. Thank you for bringing this to our attention. We have made the following revisions to include this distinction.

Methods section, page 20-21, lines 528-529
*“All patients included in the analysis had no documented history of olfactory or gustatory dysfunction prior to COVID-19 diagnosis.”*
Methods section, page 22, lines 577-581
*“Participants were included in the present study if they answered “Yes” to the question “Were you tested for COVID-19 in the past month?, “Yes” to the question “Was the test for COVID-19 positive?”, and reported their symptoms to the question “Which of the following symptoms did you have? (select all that apply).”, did not skip, and had no previous loss of smell or taste perception.”*

We did not include other nasal conditions or medications as factors in our analyses. We have similar data/participant inclusion requirements and data control methods as reported in the largest GWAS to date for this phenotype, Shelton et al. 2022 (Manuscript reference 13).

Shelton JF, Shastri AJ, Fletez-Brant K, Aslibekyan S, Auton A. The UGT2A1/UGT2A2 locus is associated with COVID-19-related loss of smell or taste. Nat Genet. 2022;54:121-124.

**Comment 4:** How were the effects of different COVID-19 variants on symptoms excluded? These issues were not adequately addressed in the paper, casting doubt on the reliability of the conclusions.

**Response:** This is a great point. We did not directly control for different COVID-19 variants in this study. With the UI cohort we did not determine the variant infecting each participant and with the AoU cohort, this data is not available. However, we can use date of data collection/survey entry as a proxy for each cohort to determine approximately which COVID-19 variants were active in the population at those times. The earlier variants of COVID-19 have a much higher rate of causing anosmia/ageusia symptoms than the omicron variant and variants after. The omicron variant was first reported on November 25^th^, 2021 and the first cases were in Africa and Asia. The latest sample collection for the UI cohort was in November 2021 and the latest survey data entry in the AoU cohort was March 2021. With all samples reported for both cohorts before the omicron variant was active in the United States population, we can say that the participants were infected by an early COVID-19 variant that have similar prevalence of anosmia/ageusia symptoms.

Thank you for this question. We have made the following revisions in the manuscript.

Discussion section, page 19, lines 491-497 *“The final main limitation with the analysis is not having the ability to control for SARS-CoV-2 infection variant/strain or including a temporal and geographical (AoU cohort) assessment of cases and controls. The omicron SARS-CoV-2 variant was first reported November 25th, 2021 and presents with lower rates of anosmia/ageusia symptoms than earlier SARS-CoV-2 variant infections [75,76,77]. Using data collection (UI cohort) and survey entry (AoU cohort) dates as proxies, all participants were infected with a SARS-CoV-2 variant that preceded omicron and exhibited similar rates of anosmia/ageusia symptoms.”*

Methods section, page 21, lines 530-531
*“All participants were recruited and samples collected before December 2021.”*
Methods section, page 23, lines 603-604
*“All participants had survey data entry dates prior to April 2021.”*

Reference section, page 38, ref # 75, 76, 76, lines 1013-1019

*75.	Karim SS, Karim QA. Omicron SARS-CoV-2 variant: a new chapter in the COVID-19 pandemic. Lancet. 2021;398: 2126-2128.*

*76.	Butowt R, Bilińska K, von Bartheld C. Why does the omicron variant largely spare olfactory function? Implications for the pathogenesis of anosmia in coronavirus disease 2019. J. Infect. Dis. 2022;226: 1304-1308.*

*77.	von Bartheld CS, Wang L. Prevalence of olfactory dysfunction with the omicron variant of SARS-CoV-2: a systematic review and meta-analysis. Cells. 2023;12: 430.*
