# Supplementary material for: Genome-wide machine learning analysis of anosmia and ageusia with COVID-19
Source: PLoS One. 2026 Aug 13;21(8):e0355832. doi: 10.1371/journal.pone.0355832 (PMC13472366; doi:10.1371/journal.pone.0355832)
Supplement: Supplementary file 8 [file pone.0355832.s042.docx]

Response to Academic Editors and Reviewers

Thank you very much for taking the time to review this manuscript and all associated documentation. Please find the detailed responses below and the corresponding revisions/corrections highlighted/in track changes in the revised manuscript files.

**Journal Requirements**:

When submitting your revision, we need you to address these additional requirements.

1. Please ensure that your manuscript meets PLOS ONE's style requirements, including those for file naming. The PLOSONE style templates can be found at

<https://journals.plos.org/plosone/s/file?id=wjVg/PLOSOne_formatting_sample_main_body.pdf> and

[https://journals.plos.org/plosone/s/file?id=ba62/PLOSOne_formatting_sample_title_authors_affiliations.pdf](https://nam12.safelinks.protection.outlook.com/?url=https%3A%2F%2Ftrack.editorialmanager.com%2FCL0%2Fhttps%3A%252F%252Fjournals.plos.org%252Fplosone%252Fs%252Ffile%253Fid%3Dba62%252FPLOSOne_formatting_sample_title_authors_affiliations.pdf%2F1%2F010f019c1c2b6b2d-98f332cd-986c-4adc-8d0e-b28011bf60ee-000000%2FTx0HUVOwOpTNNgeHJpk4NU5r8zlQ9joGxDZ2lvxVu90%3D248&data=05%7C02%7Clpietan%40uiowa.edu%7Cead00c11a2d04812efb608de6202583a%7C1bc445959aba4fc3b8ec7b94a5586fdc%7C1%7C1%7C639055959654622772%7CUnknown%7CTWFpbGZsb3d8eyJFbXB0eU1hcGkiOnRydWUsIlYiOiIwLjAuMDAwMCIsIlAiOiJXaW4zMiIsIkFOIjoiTWFpbCIsIldUIjoyfQ%3D%3D%7C0%7C%7C%7C&sdata=T8EK1YE%2FmVjX6Y%2BvygDlxqDnAb%2BeLd%2BL1shHMxNKflU%3D&reserved=0)

**Response:** We have utilized the resources in the links above to reformat our manuscript to meet PLOS ONE's style requirements.

2. Please note that PLOS One has specific guidelines on code sharing for submissions in which author-generated code underpins the findings in the manuscript. In these cases, we expect all author-generated code to be made available without restrictions upon publication of the work. Please review our guidelines at <https://journals.plos.org/plosone/s/materials-and-software-sharing#loc-sharing-code> and ensure that your code is shared in a way that follows best practice and facilitates reproducibility and reuse.

**Response:** All author-generated code used in the analysis has been uploaded and is publicly available at <https://github.com/lpietan/COVID-19>

3. If the reviewer comments include a recommendation to cite specific previously published works, please review and evaluate these publications to determine whether they are relevant and should be cited. There is no requirement to cite these works unless the editor has indicated otherwise.

**Response:** Not applicable

**Reviewer Comments to the Author**

**Reviewer #1:**

1. Phenotypic Heterogeneity: The Methods section classifies anosmia/ageusia as a binary variable. However, from a clinical perspective, transient anosmia (often driven by mucosal edema) and persistent anosmia (suggestive of neuroepithelial damage) likely involve distinct pathophysiological mechanisms. Pooling these distinct phenotypes in a small discovery cohort (UI cohort, N=187) risks diluting genetic signals and increasing phenotypic noise, which compromises the statistical power to detect true associations.

**Response:** We completely agree with this comment. The intention of the survey question (both UI and AoU cohorts) asking patients if they have experienced anosmia and/or ageusia with COVID-19 is attempting to ask about persistent anosmia/ageusia. However, a patient’s understanding of this clinical distinction when self-reporting symptoms is unknown and there is a chance of misreporting the symptoms leading to phenotypic heterogeneity. We included in the manuscript that patient self-reporting through surveys was a limitation of the study, and this could lead to noise in our analysis (Discussion, page 19, lines 476-479). We have revised this section to further clarify the limitations.

The following revision has been made to the limitations section of the discussion (page 20, lines 477-492)

*“Another limitation is with patient self-reporting of their COVID-19 symptoms through surveys with yes/no answers in the UI and AoU cohort. Asking patients in person or through surveys to assess their own symptoms without clear definition (metric-based) or complete understanding of the symptoms, potentially with significant time after symptoms subsided, leads to phenotypic heterogeneity. This could manifest as inconsistencies between cases and controls, as symptom awareness and interpretation can be influenced by an individual's condition and background, as well as their understanding of the intricacies of the anosmia and ageusia symptoms. This includes distinctions such as transient anosmia associated with mucosal edema versus persistent anosmia, and the understanding of true loss of taste versus loss of flavor due to olfactory dysfunction. The phenotypic heterogeneity adds noise to the machine learning analysis, dampening and/or biasing the genetic associations and the conclusions drawn. The binary phenotype derived from the yes/no survey responses captures a broad definition of anosmia/ageusia with COVID-19, whereas more granular, clearly defined, and metric-based phenotyping would likely yield more precise genetic associations and better resolve distinct pathophysiological mechanisms underlying these symptoms. Future studies plan to incorporate deep phenotyping and metric-based chemosensory testing to better define these phenotypes, address these limitations, and improve the resolution of our genetic association analyses.”*

2. Clinical Confusion of Taste vs. Flavor: Patients frequently confuse the loss of flavor (retronasal olfaction) with true ageusia. Since the phenotyping relied on self-reported surveys, please acknowledge in the Discussion that some "ageusia" cases may clinically represent "anosmia."

**Response:** This is another great point. Thank you. As stated in the previous response, we mentioned the limitation of patient self-reporting through survey (Discussion, page 19, lines 476-479) but have made revisions in the manuscript to state this point.

Revision have been made to the limitations section of the discussion (page 20, lines 477-492) as noted above in response to comment #1.

3. Biological Plausibility of SLC2A11: The authors state that SLC2A11 is "lowly expressed in cells obtained from the nasal cavity". Given this low expression in the target tissue, the hypothesis that this gene is a "key driver" of olfactory cilia dysfunction requires a more robust justification.

**Response:** Thank you for this comment. The intended meaning of the statement, “GLUT11 (SLC2A11) has a function of transporting glucose and fructose across the cell membrane and is lowly expressed in most cell types including cells obtained from the nasal cavity and immune cells [43,44,45].” (Discussion, page 15, lines 359-361), was that there is at least low expression of SLC2A11 in most tissue types including cells from the nasal cavity and immune cells. Even with low expression, an abnormal protein can affect the biological system(s) the protein is involved in.

The following revision has been made within the manuscript (Discussion, page 16, lines 361-363).

*“GLUT11 (SLC2A11) has a function of transporting glucose and fructose across the cell membrane and has a low to moderate baseline level of expression in most cell types including cells obtained from the nasal cavity and immune cells [43,44,45].”*

**Reviewer #2:** 
This is an interesting study applying multiple machine learning approaches to identify candidate genes and variants associated with COVID-19–related anosmia and ageusia using two independent cohorts (UI and AoU). The analytical pipeline is now clearly described, and the authors have addressed the concerns raised by previous reviewers. I have one remaining concern regarding patient phenotyping.
Although the authors acknowledge the limitation of symptom-based classification, the potential rate of misclassification inherent in self-reported smell and taste loss deserves more explicit discussion. Subjective chemosensory complaints are influenced by nasal obstruction, mood or cognitive state, and individual awareness, and therefore may not accurately reflect true olfactory or gustatory dysfunction. Importantly, such phenotypic misclassification introduces “noisy labels,” which can directly affect machine learning–based feature selection and variable importance ranking, potentially diluting or biasing genetic associations.
In addition, olfactory and gustatory dysfunction exist along a spectrum from transient to persistent loss, likely reflecting distinct underlying mechanisms. A binary yes/no symptom definition therefore likely captures a broad symptomatic phenotype rather than a strictly sensorineural one. Clarifying this distinction and its implications for model performance and biological interpretation would strengthen the manuscript.
I encourage the authors to address these issues more explicitly in the discussion and to consider how future studies incorporating objective chemosensory testing or more granular phenotyping could improve genetic inference.

**Response:** Thank you for these comments. We agree with these comments as they are similar to the previous comments discussed above. We had a brief section in the manuscript discussing the limitations of patient self-reporting through surveys (Discussion, page 15, lines 359-361) but have elaborated on the points brought up here. We have revised the manuscript to more clearly discuss the limitations and avenues for future studies to address them. As for the binary symptom definition for phenotypes, a previous study has utilized patient self-reporting through surveys to define the phenotypes of anosmia and ageusia with COVID-19. This was the largest GWAS performed to date for these phenotypes and is reference #13 in the manuscript, discussed in multiple areas. However, we agree this is still a limitation and have added revisions to discuss this point and the implications on our analysis and interpretation of the results.

Revision have been made to the limitations section of the discussion (page 20, lines 477-492) as noted above in response to comment #1 of reviewer #1.
