# Supplementary material for: Genome-wide machine learning analysis of anosmia and ageusia with COVID-19
Source: PLoS One. 2026 Aug 13;21(8):e0355832. doi: 10.1371/journal.pone.0355832 (PMC13472366; doi:10.1371/journal.pone.0355832)
Supplement: Peer review history

# Author comment: Author response to Decision Letter 0

**DOI:** 10.1371/journal.pone.0355832.r001

**Transfer Alert**

This paper was transferred from another journal. As a result, its full editorial history (including decision letters, peer reviews and author responses) may not be present.

5 Dec 2025

Attachment Submitted filename: Response to Reviewers and Editors.docx

# Peer review: Decision Letter 0

**DOI:** 10.1371/journal.pone.0355832.r002

**Authors:** Rong-San Jiang

1 Feb 2026

Dear Dr. Pietan,

Thank you for submitting your manuscript to PLOS ONE. After careful consideration, we feel that it has merit but does not fully meet PLOS ONE’s publication criteria as it currently stands. Therefore, we invite you to submit a revised version of the manuscript that addresses the points raised during the review process.

Please submit your revised manuscript by Mar 18 2026 11:59PM. If you will need more time than this to complete your revisions, please reply to this message or contact the journal office at plosone@plos.org. When you're ready to submit your revision, log on to https://www.editorialmanager.com/pone/ and select the 'Submissions Needing Revision' folder to locate your manuscript file.

- A letter that responds to each point raised by the academic editor and reviewer(s). You should upload this letter as a separate file labeled 'Response to Reviewers'.
- A marked-up copy of your manuscript that highlights changes made to the original version. You should upload this as a separate file labeled 'Revised Manuscript with Track Changes'.
- An unmarked version of your revised paper without tracked changes. You should upload this as a separate file labeled 'Manuscript'.

If you would like to make changes to your financial disclosure, please include your updated statement in your cover letter. Guidelines for resubmitting your figure files are available below the reviewer comments at the end of this letter.

If applicable, we recommend that you deposit your laboratory protocols in protocols.io to enhance the reproducibility of your results. Protocols.io assigns your protocol its own identifier (DOI) so that it can be cited independently in the future. For instructions see: https://journals.plos.org/plosone/s/submission-guidelines#loc-laboratory-protocols. Additionally, PLOS ONE offers an option for publishing peer-reviewed Lab Protocol articles, which describe protocols hosted on protocols.io. Read more information on sharing protocols at https://plos.org/protocols?utm_medium=editorial-email&utm_source=authorletters&utm_campaign=protocols.

We look forward to receiving your revised manuscript.

Kind regards,

Rong-San Jiang

Academic Editor

PLOS One

Journal Requirements:

When submitting your revision, we need you to address these additional requirements.

1. Please ensure that your manuscript meets PLOS ONE's style requirements, including those for file naming. The PLOS ONE style templates can be found at

https://journals.plos.org/plosone/s/file?id=wjVg/PLOSOne_formatting_sample_main_body.pdf and

https://journals.plos.org/plosone/s/file?id=ba62/PLOSOne_formatting_sample_title_authors_affiliations.pdf

2. Please note that PLOS One has specific guidelines on code sharing for submissions in which author-generated code underpins the findings in the manuscript. In these cases, we expect all author-generated code to be made available without restrictions upon publication of the work. Please review our guidelines at https://journals.plos.org/plosone/s/materials-and-software-sharing#loc-sharing-code and ensure that your code is shared in a way that follows best practice and facilitates reproducibility and reuse.

3. If the reviewer comments include a recommendation to cite specific previously published works, please review and evaluate these publications to determine whether they are relevant and should be cited. There is no requirement to cite these works unless the editor has indicated otherwise.

[Note: HTML markup is below. Please do not edit.]

Reviewers' comments:

Reviewer's Responses to Questions

**Comments to the Author**

1. Is the manuscript technically sound, and do the data support the conclusions?

Reviewer #1: Yes

Reviewer #2: Yes

**********

2. Has the statistical analysis been performed appropriately and rigorously?

Reviewer #1: Yes

Reviewer #2: Yes

**********

3. Have the authors made all data underlying the findings in their manuscript fully available?

The PLOS Data policy

Reviewer #1: Yes

Reviewer #2: Yes

**********

4. Is the manuscript presented in an intelligible fashion and written in standard English?

Reviewer #1: Yes

Reviewer #2: Yes

**********

Reviewer #1: 1. Phenotypic Heterogeneity: The Methods section classifies anosmia/ageusia as a binary variable. However, from a clinical perspective, transient anosmia (often driven by mucosal edema) and persistent anosmia (suggestive of neuroepithelial damage) likely involve distinct pathophysiological mechanisms. Pooling these distinct phenotypes in a small discovery cohort (UI cohort, N=187) risks diluting genetic signals and increasing phenotypic noise, which compromises the statistical power to detect true associations.

2. Clinical Confusion of Taste vs. Flavor: Patients frequently confuse the loss of flavor (retronasal olfaction) with true ageusia. Since the phenotyping relied on self-reported surveys, please acknowledge in the Discussion that some "ageusia" cases may clinically represent "anosmia."

3. Biological Plausibility of SLC2A11: The authors state that SLC2A11 is "lowly expressed in cells obtained from the nasal cavity". Given this low expression in the target tissue, the hypothesis that this gene is a "key driver" of olfactory cilia dysfunction requires a more robust justification.

Reviewer #2: This is an interesting study applying multiple machine learning approaches to identify candidate genes and variants associated with COVID-19–related anosmia and ageusia using two independent cohorts (UI and AoU). The analytical pipeline is now clearly described, and the authors have addressed the concerns raised by previous reviewers. I have one remaining concern regarding patient phenotyping.

Although the authors acknowledge the limitation of symptom-based classification, the potential rate of misclassification inherent in self-reported smell and taste loss deserves more explicit discussion. Subjective chemosensory complaints are influenced by nasal obstruction, mood or cognitive state, and individual awareness, and therefore may not accurately reflect true olfactory or gustatory dysfunction. Importantly, such phenotypic misclassification introduces “noisy labels,” which can directly affect machine learning–based feature selection and variable importance ranking, potentially diluting or biasing genetic associations.

In addition, olfactory and gustatory dysfunction exist along a spectrum from transient to persistent loss, likely reflecting distinct underlying mechanisms. A binary yes/no symptom definition therefore likely captures a broad symptomatic phenotype rather than a strictly sensorineural one. Clarifying this distinction and its implications for model performance and biological interpretation would strengthen the manuscript.

I encourage the authors to address these issues more explicitly in the discussion and to consider how future studies incorporating objective chemosensory testing or more granular phenotyping could improve genetic inference.

**********

what does this mean?). If published, this will include your full peer review and any attached files.

If you choose “no”, your identity will remain anonymous but your review may still be made public.

**Do you want your identity to be public for this peer review?**  For information about this choice, including consent withdrawal, please see our Privacy Policy

Reviewer #1: No

Reviewer #2: **Yes:**  Yun-Ting Chao

**********

[NOTE: If reviewer comments were submitted as an attachment file, they will be attached to this email and accessible via the submission site. Please log into your account, locate the manuscript record, and check for the action link "View Attachments". If this link does not appear, there are no attachment files.]

To ensure your figures meet our technical requirements, please review our figure guidelines: https://journals.plos.org/plosone/s/figures

You may also use PLOS’s free figure tool, NAAS, to help you prepare publication quality figures: https://journals.plos.org/plosone/s/figures#loc-tools-for-figure-preparation.

NAAS will assess whether your figures meet our technical requirements by comparing each figure against our figure specifications.

Attachment Submitted filename: Comments_ML_COVID_gene.docx

# Author comment: Author response to Decision Letter 1

**DOI:** 10.1371/journal.pone.0355832.r003

23 Jun 2026

Thank you very much for taking the time to review this manuscript and all associated documentation. Please find the detailed responses below and the corresponding revisions/corrections highlighted/in track changes in the revised manuscript files.

Journal Requirements:

When submitting your revision, we need you to address these additional requirements.

1. Please ensure that your manuscript meets PLOS ONE's style requirements, including those for file naming. The PLOSONE style templates can be found at

https://journals.plos.org/plosone/s/file?id=wjVg/PLOSOne_formatting_sample_main_body.pdf and

https://journals.plos.org/plosone/s/file?id=ba62/PLOSOne_formatting_sample_title_authors_affiliations.pdf

Response: We have utilized the resources in the links above to reformat our manuscript to meet PLOS ONE's style requirements.

2. Please note that PLOS One has specific guidelines on code sharing for submissions in which author-generated code underpins the findings in the manuscript. In these cases, we expect all author-generated code to be made available without restrictions upon publication of the work. Please review our guidelines at https://journals.plos.org/plosone/s/materials-and-software-sharing#loc-sharing-code and ensure that your code is shared in a way that follows best practice and facilitates reproducibility and reuse.

Response: All author-generated code used in the analysis has been uploaded and is publicly available at https://github.com/lpietan/COVID-19

3. If the reviewer comments include a recommendation to cite specific previously published works, please review and evaluate these publications to determine whether they are relevant and should be cited. There is no requirement to cite these works unless the editor has indicated otherwise.

Response: Not applicable

Reviewer Comments to the Author

Reviewer #1:

1. Phenotypic Heterogeneity: The Methods section classifies anosmia/ageusia as a binary variable. However, from a clinical perspective, transient anosmia (often driven by mucosal edema) and persistent anosmia (suggestive of neuroepithelial damage) likely involve distinct pathophysiological mechanisms. Pooling these distinct phenotypes in a small discovery cohort (UI cohort, N=187) risks diluting genetic signals and increasing phenotypic noise, which compromises the statistical power to detect true associations.

Response: We completely agree with this comment. The intention of the survey question (both UI and AoU cohorts) asking patients if they have experienced anosmia and/or ageusia with COVID-19 is attempting to ask about persistent anosmia/ageusia. However, a patient’s understanding of this clinical distinction when self-reporting symptoms is unknown and there is a chance of misreporting the symptoms leading to phenotypic heterogeneity. We included in the manuscript that patient self-reporting through surveys was a limitation of the study, and this could lead to noise in our analysis (Discussion, page 19, lines 476-479). We have revised this section to further clarify the limitations.

The following revision has been made to the limitations section of the discussion (page 20, lines 477-492)

“Another limitation is with patient self-reporting of their COVID-19 symptoms through surveys with yes/no answers in the UI and AoU cohort. Asking patients in person or through surveys to assess their own symptoms without clear definition (metric-based) or complete understanding of the symptoms, potentially with significant time after symptoms subsided, leads to phenotypic heterogeneity. This could manifest as inconsistencies between cases and controls, as symptom awareness and interpretation can be influenced by an individual's condition and background, as well as their understanding of the intricacies of the anosmia and ageusia symptoms. This includes distinctions such as transient anosmia associated with mucosal edema versus persistent anosmia, and the understanding of true loss of taste versus loss of flavor due to olfactory dysfunction. The phenotypic heterogeneity adds noise to the machine learning analysis, dampening and/or biasing the genetic associations and the conclusions drawn. The binary phenotype derived from the yes/no survey responses captures a broad definition of anosmia/ageusia with COVID-19, whereas more granular, clearly defined, and metric-based phenotyping would likely yield more precise genetic associations and better resolve distinct pathophysiological mechanisms underlying these symptoms. Future studies plan to incorporate deep phenotyping and metric-based chemosensory testing to better define these phenotypes, address these limitations, and improve the resolution of our genetic association analyses.”

2. Clinical Confusion of Taste vs. Flavor: Patients frequently confuse the loss of flavor (retronasal olfaction) with true ageusia. Since the phenotyping relied on self-reported surveys, please acknowledge in the Discussion that some "ageusia" cases may clinically represent "anosmia."

Response: This is another great point. Thank you. As stated in the previous response, we mentioned the limitation of patient self-reporting through survey (Discussion, page 19, lines 476-479) but have made revisions in the manuscript to state this point.

Revision have been made to the limitations section of the discussion (page 20, lines 477-492) as noted above in response to comment #1.

3. Biological Plausibility of SLC2A11: The authors state that SLC2A11 is "lowly expressed in cells obtained from the nasal cavity". Given this low expression in the target tissue, the hypothesis that this gene is a "key driver" of olfactory cilia dysfunction requires a more robust justification.

Response: Thank you for this comment. The intended meaning of the statement, “GLUT11 (SLC2A11) has a function of transporting glucose and fructose across the cell membrane and is lowly expressed in most cell types including cells obtained from the nasal cavity and immune cells [43,44,45].” (Discussion, page 15, lines 359-361), was that there is at least low expression of SLC2A11 in most tissue types including cells from the nasal cavity and immune cells. Even with low expression, an abnormal protein can affect the biological system(s) the protein is involved in.

The following revision has been made within the manuscript (Discussion, page 16, lines 361-363).

“GLUT11 (SLC2A11) has a function of transporting glucose and fructose across the cell membrane and has a low to moderate baseline level of expression in most cell types including cells obtained from the nasal cavity and immune cells [43,44,45].”

Reviewer #2:

This is an interesting study applying multiple machine learning approaches to identify candidate genes and variants associated with COVID-19–related anosmia and ageusia using two independent cohorts (UI and AoU). The analytical pipeline is now clearly described, and the authors have addressed the concerns raised by previous reviewers. I have one remaining concern regarding patient phenotyping.

Although the authors acknowledge the limitation of symptom-based classification, the potential rate of misclassification inherent in self-reported smell and taste loss deserves more explicit discussion. Subjective chemosensory complaints are influenced by nasal obstruction, mood or cognitive state, and individual awareness, and therefore may not accurately reflect true olfactory or gustatory dysfunction. Importantly, such phenotypic misclassification introduces “noisy labels,” which can directly affect machine learning–based feature selection and variable importance ranking, potentially diluting or biasing genetic associations.

In addition, olfactory and gustatory dysfunction exist along a spectrum from transient to persistent loss, likely reflecting distinct underlying mechanisms. A binary yes/no symptom definition therefore likely captures a broad symptomatic phenotype rather than a strictly sensorineural one. Clarifying this distinction and its implications for model performance and biological interpretation would strengthen the manuscript.

I encourage the authors to address these issues more explicitly in the discussion and to consider how future studies incorporating objective chemosensory testing or more granular phenotyping could improve genetic inference.

Response: Thank you for these comments. We agree with these comments as they are similar to the previous comments discussed above. We had a brief section in the manuscript discussing the limitations of patient self-reporting through surveys (Discussion, page 15, lines 359-361) but have elaborated on the points brought up here. We have revised the manuscript to more clearly discuss the limitations and avenues for future studies to address them. As for the binary symptom definition for phenotypes, a previous study has utilized patient self-reporting through surveys to define the phenotypes of anosmia and ageusia with COVID-19. This was the largest GWAS performed to date for these phenotypes and is reference #13 in the manuscript, discussed in multiple areas. However, we agree this is still a limitation and have added revisions to discuss this point and the implications on our analysis and interpretation of the results.

Revision have been made to the limitations section of the discussion (page 20, lines 477-492) as noted above in response to comment #1 of reviewer #1.

Attachment Submitted filename: Response to Reviewers.docx

# Peer review: Decision Letter 1

**DOI:** 10.1371/journal.pone.0355832.r004

**Authors:** Rong-San Jiang

28 Jul 2026

Genome-wide machine learning analysis of anosmia and ageusia with COVID-19

PONE-D-25-61396R1

Dear Dr. Pietan,

We’re pleased to inform you that your manuscript has been judged scientifically suitable for publication and will be formally accepted for publication once it meets all outstanding technical requirements.

Within one week, you’ll receive an e-mail detailing the required amendments. When these have been addressed, you’ll receive a formal acceptance letter and your manuscript will be scheduled for publication.

An invoice will be generated when your article is formally accepted. Please note, if your institution has a publishing partnership with PLOS and your article meets the relevant criteria, all or part of your publication costs will be covered. Please make sure your user information is up-to-date by logging into Editorial Manager at Editorial Manager® and clicking the ‘Update My Information' link at the top of the page. For questions related to billing, please contact billing support.

If your institution or institutions have a press office, please notify them about your upcoming paper to help maximize its impact. If they’ll be preparing press materials, please inform our press team as soon as possible -- no later than 48 hours after receiving the formal acceptance. Your manuscript will remain under strict press embargo until 2 pm Eastern Time on the date of publication. For more information, please contact onepress@plos.org.

Kind regards,

Rong-San Jiang

Academic Editor

PLOS One

Additional Editor Comments (optional):

Reviewers' comments:

Reviewer's Responses to Questions

**Comments to the Author**

Reviewer #1: All comments have been addressed

Reviewer #2: All comments have been addressed

**********

2. Is the manuscript technically sound, and do the data support the conclusions?

Reviewer #1: Yes

Reviewer #2: Yes

**********

3. Has the statistical analysis been performed appropriately and rigorously?

Reviewer #1: Yes

Reviewer #2: Yes

**********

4. Have the authors made all data underlying the findings in their manuscript fully available?

The PLOS Data policy

Reviewer #1: Yes

Reviewer #2: Yes

**********

5. Is the manuscript presented in an intelligible fashion and written in standard English?

Reviewer #1: Yes

Reviewer #2: Yes

**********

Reviewer #1: (No Response)

Reviewer #2: The authors have addressed all my comments well. However, this work should still be interpreted with caution, given that the selection for anosmia/ageusia is based on a subjective binary response from patients.

**********

what does this mean?). If published, this will include your full peer review and any attached files.

If you choose “no”, your identity will remain anonymous but your review may still be made public.

**Do you want your identity to be public for this peer review?**  For information about this choice, including consent withdrawal, please see our Privacy Policy

Reviewer #1: No

Reviewer #2: No

**********

# Editor report: Acceptance letter

**DOI:** 10.1371/journal.pone.0355832.r005

**Authors:** Rong-San Jiang

PONE-D-25-61396R1

PLOS One

Dear Dr. Pietan,

I'm pleased to inform you that your manuscript has been deemed suitable for publication in PLOS One. Congratulations! Your manuscript is now being handed over to our production team.

At this stage, our production department will prepare your paper for publication. This includes ensuring the following:

* All references, tables, and figures are properly cited

* All relevant supporting information is included in the manuscript submission,

* There are no issues that prevent the paper from being properly typeset

You will receive further instructions from the production team, including instructions on how to review your proof when it is ready. Please keep in mind that we are working through a large volume of accepted articles, so please give us a few days to review your paper and let you know the next and final steps.

Lastly, if your institution or institutions have a press office, please let them know about your upcoming paper now to help maximize its impact. If they'll be preparing press materials, please inform our press team within the next 48 hours. Your manuscript will remain under strict press embargo until 2 pm Eastern Time on the date of publication. For more information, please contact onepress@plos.org.

You will receive an invoice from PLOS for your publication fee after your manuscript has reached the completed accept phase. If you receive an email requesting payment before acceptance or for any other service, this may be a phishing scheme. Learn how to identify phishing emails and protect your accounts at https://explore.plos.org/phishing.

If we can help with anything else, please email us at customercare@plos.org.

Thank you for submitting your work to PLOS ONE and supporting open access.

Kind regards,

PLOS ONE Editorial Office Staff

on behalf of

Dr. Rong-San Jiang

Academic Editor

PLOS One
